# Single-cell profiling of T cells uncovers a tissue-resident memory-like T-cell subset associated with bidirectional prognosis for B-cell acute lymphoblastic leukemia

**DOI:** 10.3389/fimmu.2022.957436

**Published:** 2022-12-02

**Authors:** Wenpu Lai, Xiaofang Wang, Lian Liu, Ling Xu, Lipeng Mao, Jiaxiong Tan, Xianfeng Zha, Huien Zhan, Wen Lei, Yu Lan, Guobing Chen, Yangqiu Li, Oscar Junhong Luo

**Affiliations:** ^1^ Department of Hematology, First Affiliated Hospital, Jinan University, Guangzhou, China; ^2^ Key Laboratory for Regenerative Medicine of Ministry of Education, Institute of Hematology, School of Medicine, Jinan University, Guangzhou, China; ^3^ Department of Systems Biomedical Sciences, School of Medicine, Jinan University, Guangzhou, China; ^4^ Department of Hematology/Oncology, Guangzhou Women and Children’s Medical Center, Guangzhou Medical University, Guangzhou, Guangdong, China; ^5^ Department of Microbiology and Immunology, School of Medicine, Institute of Geriatric Immunology, School of Medicine, Jinan University, Guangzhou, China; ^6^ Department of Clinical Laboratory, First Affiliated Hospital, Jinan University, Guangzhou, China

**Keywords:** hematologic neoplasms, immunity, T-lymphocytes, immunologic memory, gene expression profiling

## Abstract

**Introduction:**

The character and composition of leukemia-related T cells are closely related to the treatment response and prognosis for patients. Though B cell-acute lymphoblastic leukemia (B-ALL) patients have benefited from immune-based approaches, such as chimeric antigen receptor T cells therapy, some of them still end with poor prognosis, especially for adult patients. Therefore, deep understanding of the developmental relationship between T cell subtypes in relation to B-ALL patient prognosis is urgently needed.

**Methods:**

We analyzed the peripheral blood T cell single-cell RNA sequencing data of three B-ALL patients, using data from 11 healthy individuals as controls. In total, 16,143 and 53,701 T cells from B-ALL patients and healthy adults, respectively, were objectively analyzed for detailed delineation of 13 distinct T cell clusters. Cluster-specific genes were used as marker genes to annotate each T cell subtype.

**Results:**

Unbiased analysis enabled the discovery of circulating CD103+ T cell (CD3+CD103+MKI67+), also defined as tissue-resident memory-like T (Trm-like) cell, populations were elevated in B-ALL patients, which expressed high level of cell proliferation and exhaustion related genes. In addition, cell fate trajectory analysis showed these Trm-like cells, which shared T-cell receptor (TCR) clonotypes with exhausted T (Tex) cells and effector T (Teff) cells, were supposed to transition into Teff cells; however, mainly transformed into Tex cells in leukemia environment. More importantly, Trm-like cells transformation into Teff cells and Tex cells potentially led to favorable or poor prognosis for B-ALL patients, respectively.

**Conclusion:**

In sum, a circulating Trm-like cell subset with high level expression of cell proliferation and exhaustion related genes was elevated in B-ALL patients. The bidirectional developmental potential of these T cells into Teff or Tex is closely associated with favorable or poor prognosis, respectively. Together, our study provided a unique insight of alteration of leukemia related T cells, also showed a potential immunotherapy direction and prognosis assessment model for B-ALL patients.

## Introduction

B-cell acute lymphoblastic leukemia (B-ALL) is a hematopoietic malignancy characterized by abnormal proliferation of B-lymphoid progenitor cells throughout the blood system. Analyses from the SEER (Surveillance, Epidemiology, and End Results Program) database have shown that the 5-year overall survival (OS) rate of B-ALL in children is about 89% (1). However, this rate for adult patients remains low at approximately 20%–40% (2). Prognostic factors of B-ALL include various disease-related and patient-specific factors. For example, clinical characteristics, e.g., age, white blood cell count at diagnosis, cytogenetics, and response to chemotherapy have been identified to be of prognostic significance in patients with B-ALL (3). Recently, increasing research has shown that the composition and dysfunction of immune cells are correlated with clinical treatment and prognosis in cancer patients (4–6).

Besides conventional chemotherapy and allogeneic hematopoietic stem cell transplantation, immunotherapy is surging in the last few years for fighting B-ALL (7). Novel immunotherapies comprise immune checkpoint inhibitors, tumor-targeting monoclonal antibodies, bispecific T-cell engager, and chimeric antigen receptor T-cell therapy (8). These strategies all require a deep understanding of the alteration of the host immune system, especially the T-cell immunity. Furthermore, growing experience revealed that the properties of T cells could be robust prognostic factors for disease risk and outcome in leukemia (9, 10).

In our previous work (11), we depicted different T-cell subtypes and found an exhausted cluster that specifically existed in B-ALL patients and possessed remarkable heterogeneity. However, the developmental relationship between T-cell subtypes in relation to B-ALL patient prognosis remains elusive. To address this question, further analysis was performed on these T cells in B-ALL. A subset of T cells with features of tissue-resident memory T (Trm) cells was found in the peripheral blood of B-ALL patients, which we defined as circulating CD103+ T cell or tissue-resident memory-like T (Trm-like) cell. These Trm-like cells expressing high levels of immune checkpoint molecules and cytotoxicity markers also shared similar TCR signatures with exhausted T (Tex) cells and effector T (Teff) cells. The traditional view thought that Trm cells were low in migration and long-term survival in peripheral tissues; however, more recent studies indicated that upon secondary antigen presentation, Trm cells can rejoin the circulation pool, transforming into Teff cells (12, 13). Through cell fate trajectory analysis, we found that Trm-like cells that were supposed to transit into Teff cells, however, mainly transformed into Tex cells in leukemia environment. Furthermore, Trm-like cell to Tex cell transformation potentially led to poor prognosis of B-ALL patients, while transition toward Teff cells would likely improve prognosis. Our data suggest that Trm-like cells contribute to B-ALL immunosurveillance and may provide valuable prognostic information. Further understanding of the development, maintenance, and regulation of Trm-like cells would be crucial for successful immunotherapeutic development in B-ALL.

## Methods

### Single-cell RNA sequencing datasets

The single-cell RNA sequencing (scRNA-seq) datasets of peripheral blood T cells were acquired from the Gene Expression Omnibus (GEO) database (GSE172158 and GSE157007). GSE172158 contains scRNA-seq data of peripheral blood T cells from three newly diagnosed and untreated B-ALL patients and two healthy individuals, while GSE157007 contains scRNA-seq data of peripheral blood mononuclear cells (PBMCs) from nine healthy individuals aged between 23 and 100 years. All samples except h1 of GSE172158 had matching single-cell TCR sequencing data. The information about cell preparation, scRNA-seq, TCR profiling, and quality control of GSE172158 and GSE157007 can be found from the original publications (11, 14). The GEO database accession numbers of all of these samples with other detailed information are listed in **Supplementary Table S1**.

### Quality control, data processing, and determination of cell types

scRNA-seq raw data quality control was performed to filter out low-quality cells and low-expression genes. Cells with less than 200 detected genes were removed. Meanwhile, cells with more than 15% of reads mapped to mitochondrial genes were removed. Moreover, only genes expressed in more than 10 cells were kept. Then, for the GSE157007 dataset, we removed potential doublets in each sample by using R package “DoubletFinder” (version 2.0.3) (15). After quality control, downstream analyses were performed using R package “Seurat” (version 4.0.4) (16). scRNA-seq data were normalized using the Seurat “NormalizedData” function with default parameters. Highly variable genes were identified with parameters “selection.method = vst” and “nfeatures = 2000” using the “FindVariableFeatures” function. Then, these were scaled by performing the “ScaleData” function. The “RunPCA” function was performed for dimension reduction analysis, and the “ElbowPlot” function helped to select suitable dimensionality. Different resolution parameters for unsupervised clustering were tested to find the best numbers of clusters. Non-linear dimensional reduction was performed by the “RunUMAP” function. Batch effects were removed by using the “RunHarmony” function of R package “HARMONY” (version 0.1.0) (17) before the clustering analysis in Seurat. In total, 84,219 PBMCs were annotated as different major cell types based on their average gene expression of well-known marker genes, including T cell (*CD3E*, *CD3D*, *CD3G*), B cell (*MS4A1*, *CD19*, *CD79A*), natural killer cell (*FCGR3A*, *NKG7*), monocyte and dendritic cell (*LYZ*, *CD14*, *FCER1A*), platelet (*PPBP*, *PF4*), and erythrocyte (*HBA1*, *HBA2*). Next, the T-cell cluster was placed in a subset using the Seurat “subset” function. The average expression value of *CD3D*, *CD3E*, *CD3G*, and *CD247* genes was calculated to infer the average expression level of *CD3*, and only cells of the T-cell cluster with the average expression of *CD3* greater than 0.5 were merged with two healthy individual samples from GSE172158 dataset using the “merge” function. After that, 16,143 and 53,701 high-quality T cells of B-ALL patients and healthy individuals were acquired and analyzed using “Seurat” and “HARMONY” packages as above, respectively. According to the expression of marker genes and auxiliary annotation with reference to the “MonacoImmuneData” dataset through “SingleR” (version 1.6.1) (18, 19), T cells were grouped into 13 and 11 cell types from B-ALL patients and healthy individuals, respectively. Finally, we used the Seurat4 integration method to integrate the scRNA-seq data of B-ALL patients and healthy individuals.

### Calculation of the functional gene module score

To evaluate the potential functions of interest for cell clusters, the enrichment scores of the functional gene modules were calculated by using the “AddModuleScore” function in “Seurat” at the single-cell level. The average expression levels of the corresponding cluster or group were subtracted by the aggregated expression of control gene sets. The functional modules included genes for inferring cell proliferation, cytotoxicity, and exhaustion scores. The gene sets for the functional module score calculation are listed in **Supplementary Table S2**.

### Pathway enrichment analysis

Gene Ontology (GO) and Kyoto Encyclopedia of Genes and Genomes (KEGG) enrichment analysis was conducted using R package “clusterProfiler” (version 4.0.5) (20). The “FindMarkers” function of Seurat with parameters “min.pct = 0.50” and “logfc.threshold = 0.50” was used to identify the upregulated genes in Trm-like cells of B-ALL patients in comparison to healthy individuals. Then, the “enrichGO” and “enrichKEGG” functions were used for pathway enrichment analysis of these genes. Gene symbols were converted using the “bitr” function before pathway enrichment if necessary. Full results of the pathway enrichment analysis were listed in **Supplementary Tables S3 and S4**.

### T-cell receptor repertoire analysis

T-cell clonotype data were integrated with the T-cell phenotype based on the shared cell barcodes. Clonotypes that are shared by more than or equal to two cells were defined as “Clonal,” while clonotypes identified in only one cell were defined as “Non-clonal.” T cells without a TCR clonotype detected due to sequencing limitations were defined as “Non-TCR detected.” R package “STARTRAC” (version 0.1.0) (21) was used to assess the enrichment of TCR in each cell type from the CD4+ and CD8+ T-cell subset. The degree of clonal expansion and cell fate state transition of T-cell clusters upon TCR tracking were determined using three “STARTRAC” indices, “Expansion,” “Transition,” and “pIndex.tran.”

### Reconstructing cell development trajectories

To explore the developmental progression of the selected CD4+ and CD8+ T-cell subset, R package “Monocle” (version 2.20.0) (22) was used for reconstructing their development trajectories. In detail, the raw counts for cells in each cell type were extracted and normalized by the “estimateSizeFactors” and “estimateDispersions” functions with the default parameters. Then, the “differentialGeneTest” function was used to select the top 1,000–2,000 significant genes (ordered by Q value) of CD4+ and CD8+ T-cell subset, respectively, for cell fate trajectory reconstruction. After trajectory reconstruction, the top 100 genes that had the most significantly correlated (or anti-correlated) expression profile (ordered by Q value) to the trajectory branch state were selected using the “BEAM” function. Differential genes between the three branch states were placed into three groups by the expression pattern using the ward.D2 clustering algorithm (23). Meanwhile, GO enrichment analyses were performed on genes in different clusters. Finally, the expression heatmap of the top 100 genes correlated (or anti-correlated) to the T-cell fate pseudotime was visualized using the “plot_genes_branched_heatmap” function. The top 100 genes to the T-cell fate pseudotime of the selected CD4+ and CD8+ T-cell subset were listed in **Supplementary Table S5**.

### Gene signature generation and survival analysis

To examine the role of each trajectory branch state as markers for B-ALL patient prognosis, specific gene signatures of each state were derived. Differentially expressed genes (DEGs) within each trajectory branch state were identified using the Seurat “FindAllMarkers” function. DEGs with expression having an absolute value of average log2 fold change ≥1 and being detected in more than 50% of cells were included in enrichment calculations. All DEGs used for the enrichment calculations were listed in **Supplementary Table S6**. Then, we assessed the enrichment of each state transcriptional signature through the Binding Association with Sorted Expression (BASE) algorithm (24–26) based on those DEGs. The BASE algorithm allows the generation of cell subpopulation enrichment scores in which a higher score is indicative of higher enrichment for a given cell signature. Moreover, the enrichment of each trajectory branch state in a patient was calculated by using BASE algorithm on the bulk-cell RNA-seq data from the corresponding individuals from the B-ALL dataset in the Therapeutically Applicable Research to Generate Effective Treatments (TARGET) (https://ocg.cancer.gov/programs/target) database. Patients were stratified into high and low level based on the median of the transcriptional signatures for each trajectory branch state. Survival analysis between B-ALL patients were assessed by Kaplan–Meier using R package “survival” (version 3.2-13). Results of the survival analysis were visualized using R package “survminer” (version 0.4.9).

### Integrated analysis of the pan-cancer datasets

We reanalyzed scRNA-seq data of tumor-infiltrating leukocytes (TILs) by using the Python package “SCALEX” (27) from multiple cancers (GEO accession numbers: GSE130116, GSE116256, GSE124310, GSE169246, GSE108989, GSE98638, GSE140228, GSE162025, GSE99254, GSE155698, PRJNA705464, and GSE145281) (21, 28–37).

### Flow cytometry

Flow cytometry was performed for three newly diagnosed and untreated B-ALL patients and three healthy individuals. The clinical information of these samples is listed in **Supplementary Table S7**. Cell surface staining and intracellular staining were carried out in 1× phosphate buffered saline (PBS) and using the BD Transcription Factor Buffer Set, respectively. Flow cytometry was performed using the BD LSRFortessa cytometer, and data analysis was performed using FlowJo (version 10.5.3) (BD). Cell surface and intracellular staining was performed using the following fluorophore-conjugated antibodies: CD45-BUV395 (clone HI30, BD), CD3-AF700 (clone UCHT1, BD), CD4-APC-H7 (clone RPAT4, BD), CD8-Percp-cy5.5 (clone SK1, BioLegend), Ki-67-PE-CF594 (clone Ki-67, BioLegend), PD-1-BV421 (clone MIH4, BD), and CD103-BB515 (clone Ber-ACT8, BD). Cell surface and intracellular staining was performed according to the manufacturer’s instructions.

### Statistics analysis

All statistical analyses were performed using R (version 4.1.0), including Wilcoxon rank sum test, chi-square test, and Kruskal–Wallis test. *P* < 0.05 was considered as statistically significant.

### Code availability

Analytical scripts and codes are available upon request from the corresponding authors.

## Results

### Delineation of T cells uncovers enlarged circulating CD103+ T (Trm-like) cell population in B-ALL patients

**Figure 1:**
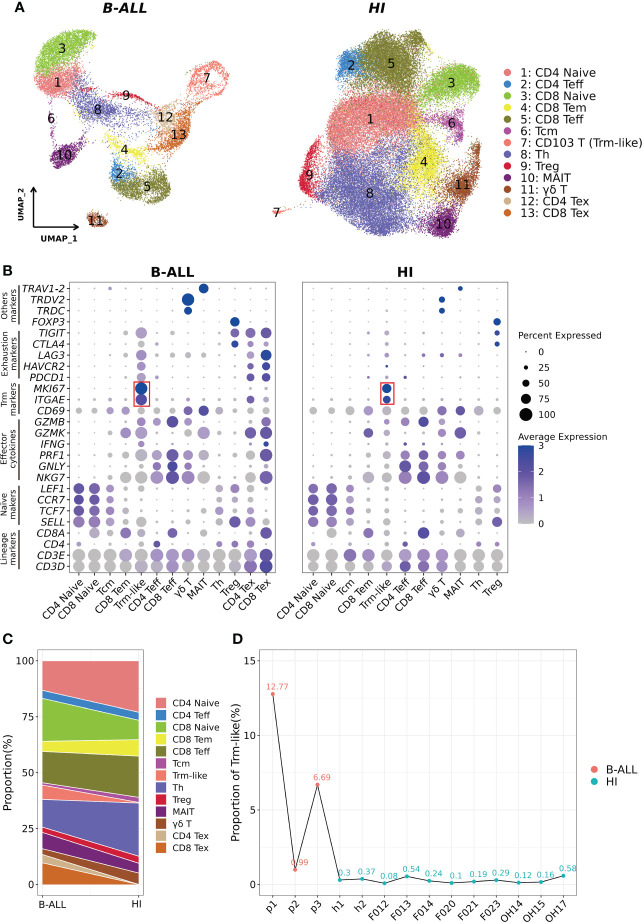
Identification of CD103+ T (Trm-like) cells in the peripheral blood of B-ALL patients and healthy individuals through scRNA-seq. **(A)**. UMAP (uniform manifold approximation and projection) visualization of T-cell single cell clusters from B-ALL patients (left) and healthy individuals (HI; right). Different clusters are depicted with distinct colors. **(B)**. Dot plot of marker genes for each cell cluster. Color scale indicates the mean of normalized expression of marker genes in each cell type, and dot size is proportional to the percentage of cells within each cell cluster expressing the marker genes. The red box highlights the marker genes for Trm-like cells. **(C)**. Variation of the proportion of each defined cell type between sample groups (B-ALL *vs*. HI). **(D)**. Proportion of Trm-like cells in each assayed sample. Red and green dots represent B-ALL patients and healthy individuals, respectively. Trm-like, tissue-resident memory-like T cell; B-ALL, B cell-acute lymphoblastic leukemia; Teff, effector T cell; Tem, effector memory T cell; Tcm, central memory T cell; Th, T-helper cell; Treg, regulatory T cell; MAIT, mucosal-associated invariant T cell; Tex, exhausted T cell.

We analyzed the T-cell scRNA-seq data of three B-ALL patients, with data from 11 healthy individuals as controls (11, 14) (*Methods*; **Supplementary Figures S1A–D**). The analyzed scRNA-seq dataset included 16,143 and 53,701 T cells from B-ALL patients and healthy individuals, respectively. Clustering of the T cells from B-ALL and healthy individuals grouped separately, and cell cluster annotation based on the expression of T-cell marker genes classified the cells into 13 distinct T-cell subtypes (**Figures 1A, B**; **Supplementary Figures S1E**, **S2A–E**). In detail, two naive T-cell clusters (CD4+ Naive and CD8+ Naive) were characterized by the high expression of *SELL*, *TCF7*, *CCR7*, and *LEF1* (38). Two Teff cell clusters (CD4+ Teff and CD8+ Teff) were defined by low expression of naive T-cell markers and high expression of effector cytokines (*NKG7*, *GNLY*, *PRF1*, and *GZMB*). Two cell populations with low naive and effector cytokine expression were defined as central memory T cells (Tcm) and CD8+ effector memory T (CD8+ Tem) cells, respectively. T-helper cell cluster (Th) was not further subdivided due to transcriptome similarity. For regulatory T cell (Treg), mucosal-associated invariant T cell (MAIT), and γδ T-cell clusters, we used *FOXP3*, *TRAV1-2*, and *TRDC* and *TRDV2* as their marker genes, respectively (39–41). Two Tex cell clusters (CD4+ Tex and CD8+ Tex) in B-ALL patients were identified based on cell exhaustion markers *PDCD1*, *LAG3*, *HAVCR2*, *CTLA4*, and *TIGIT* (42, 43). In addition, we also examined Tex cell clusters in the peripheral blood and tumors of non-leukemia cancer patients. The results showed that Tex in peripheral blood was close to 0; however, the Tex cells among the tumor-infiltrating T cells of solid tumors were similar to the peripheral blood of B-ALL (13.3% on average) (**Figure 1C**; **Supplementary Figure S2F**) (21, 28, 29). This suggests that Tex cells are only typically present in the cancerous microenvironment.

Most importantly, using *ITGAE* (*CD103*) and *MKI67* as markers, clusters of cells were identified as circulating CD103+ T (Trm-like) cells in B-ALL patients and healthy individuals, respectively (12, 44, 45), which were not previously characterized as such (11, 14). We then compared the proportion of each T-cell subtype in B-ALL and HI groups, and it was evident that B-ALL patients had significantly larger Trm-like cell population than healthy individuals, both collectively (6.39% *vs*. 0.28%) and per individual (6.82% ± 0.059% *vs*. 0.27% ± 0.0017%) (**Figures 1C, D** **)**. In addition, multicolor flow cytometry detected larger CD103+ T-cell populations in the peripheral blood samples of three additional B-ALL patients (2.33% on average) compared to three healthy individuals (0.92% on average) (**Supplementary Figures S3A–C**), which corroborated the results of our scRNA-seq analysis. Similar to B-ALL, we also found that this population of T cells highly expressing *ITGAE* and *MKI67* was elevated in the tumor microenvironment of multiple solid tumors (**Supplementary Figures S4A–C**).

### Functional characterization of Trm-like cells in the leukemia environment

**Figure 2:**
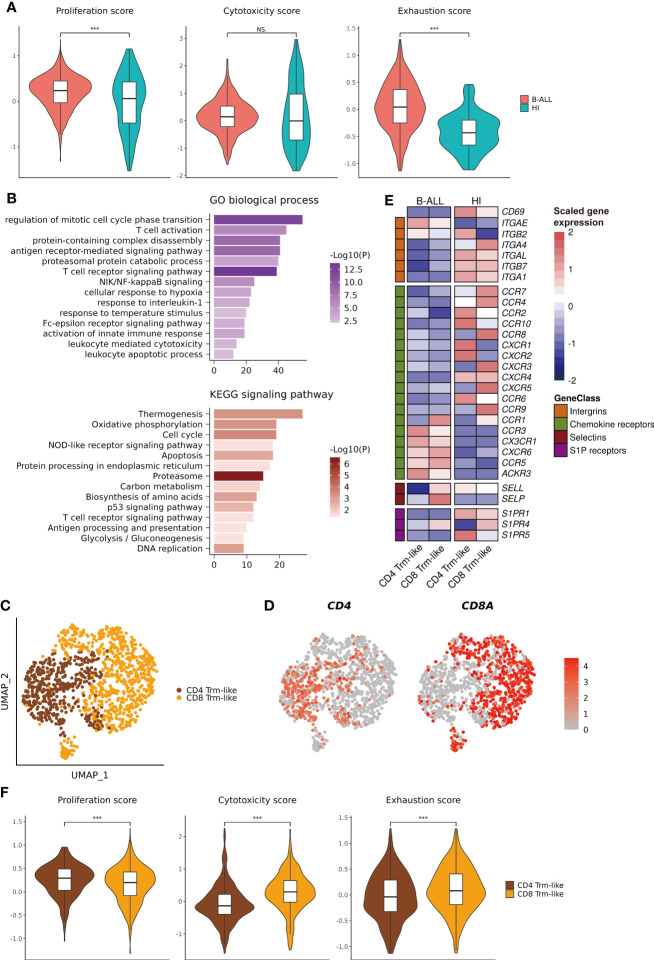
Functional characterization of Trm-like cells. **(A).** Single-cell transcriptome-derived Trm-like cell proliferation, cytotoxicity, and exhaustion score comparison between B-ALL patients and healthy individuals. **(B).** GO biological process (upper) and KEGG signaling pathway (lower) enrichment of upregulated genes in Trm-like cells of B-ALL patients in comparison to healthy individuals. **(C).** UMAP visualization of CD4+ and CD8+ Trm-like cells after reclustering of Trm-like cells from B-ALL patients and healthy individuals. **(D).** Projection of *CD4* and *CD8A* expression level on Trm-like cells from B-ALL patients and healthy individuals. **(E).** Heatmap visualization of the expression of cell migration and chemotaxis-related genes in CD4+ and CD8+ Trm-like cells from B-ALL patients and healthy individuals. **(F).** Single-cell transcriptome-derived proliferation, cytotoxicity, and exhaustion score comparison between CD4+ and CD8+ Trm-like cells from B-ALL patients. For boxplots, the outlines of the boxes represent the first and third quartiles. The line inside each box represents the median, and boundaries of the whiskers are found within the 1.5× IQR value. ***P < 0.001; NS, not significant. Wilcoxon rank sum test (two-sided).

To explore the differences in the function of Trm-like cells between B-ALL patients and healthy individuals, we calculated three functional enrichment scores (proliferation, cytotoxicity, and exhaustion) according to the expression of four corresponding gene sets (**Supplementary Table S2**; *Methods*). The results indicated that these Trm-like cells from B-ALL had significantly higher proliferation (*P* < 0.001) and exhaustion (*P* < 0.001) than HI, and there was no statistical difference in cytotoxicity between the two groups (**Figure 2A**). Meanwhile, we performed DEG analysis and GO Biological Process and KEGG signaling pathway enrichment analysis for the upregulated genes in B-ALL Trm-like cells in comparison to those of healthy individuals (**Supplementary Tables S3, S4**). The upregulated genes in B-ALL Trm-like cells included proliferation-related genes, *POLA2*, *MCM2*, and *TFDP1*, exhaustion-related genes, *PDCD1*, *LAG3*, *HAVCR2*, *TIGIT*, and *TOX*, and T-cell activation-related genes, *CSK* and *CD27*. The GO and KEGG enrichment results showed that the enriched functions and signaling pathways were related to both cell proliferation and apoptosis, such as “regulation of mitotic cell cycle phase transition,” “leukocyte apoptotic process,” “Cell cycle,” “Apoptosis,” and “DNA replication” (**Figure 2B**). Flow cytometry validation also revealed a higher percentage of Ki-67+CD103+ T cells from three additional B-ALL patients compared to healthy individuals (**Supplementary Figure S3C**). These results potentially implied that the Trm-like cells in the leukemia microenvironment were in a complex state, actively proliferating but prone to apoptosis, which were similar to the state of Trm-like cells in the solid tumor microenvironment (46). Furthermore, Trm-like cells of B-ALL patients were of genes upregulated in “Oxidative phosphorylation,” “T cell activation,” “activation of innate immune response,” and “T cell receptor signaling pathway” (**Figure 2B**), which suggested that Trm-like cells were active and involved in first-line immunity against tumors (47).

Next, we reclustered the 1,184 Trm-like cells from B-ALL patients and healthy individuals into two subtypes, CD4+ and CD8+ Trm-like cells, according to the expression of *CD4* and *CD8A* (**Figures 2C, D** **)**. Compared to Trm-like cells of healthy individuals, the DEG analysis revealed a high expression of chemokine receptors (*CCR1*, *CCR3*, *CX3CR1*, *CXCR6*, *CCR5*, and *ACKR3*) and selectins (*SELP*) and low expression of integrins (*CD69*, *ITGA4*, *ITGAL*, *ITGB7*, and *ITGA1*) and sphingosine-1-phosphate (S1P) receptors (*S1PR1* and *S1PR5*) in both CD4+ and CD8+ Trm-like cells of B-ALL patients, which were responsible for the regulation of T-cell migration (48) (**Figure 2E**). Finally, we compared the proliferation, cytotoxicity, and exhaustion scores between CD4+ and CD8+ Trm-like cells from B-ALL patients. The results indicated that compared to CD4+ Trm-like cells, CD8+ Trm-like cells had higher cytotoxicity (*P* < 0.001) and exhaustion (*P* < 0.001) scores but a lower proliferation (*P* < 0.001) score (**Figure 2F**). This potentially suggests that CD8+ Trm-like cells might play a stronger anti-leukemia effect (6, 47).

### Lineage transition tracing of T cells in B-ALL patients by TCRαβ clonotypes

**Figure 3:**
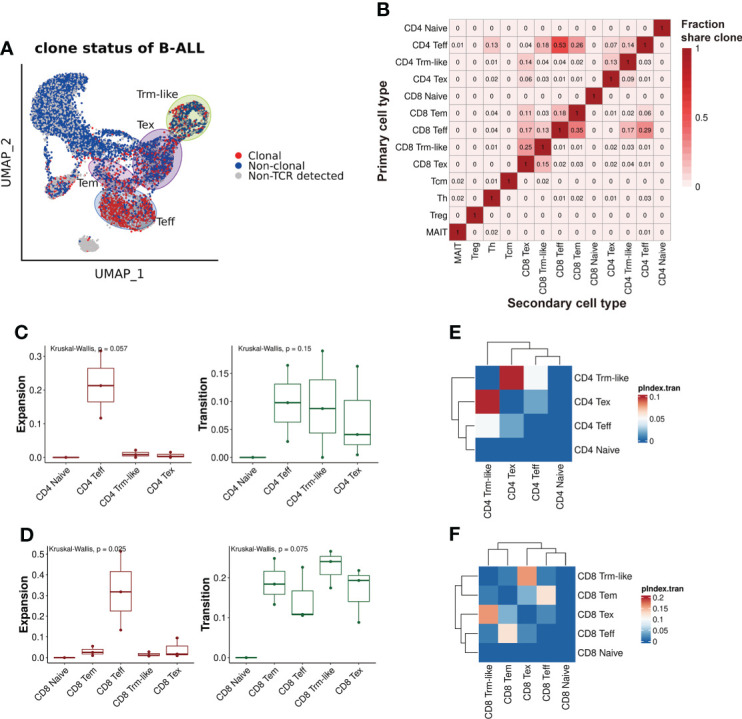
TCRαβ clonotype characterization of T cells from B-ALL patients. **(A)** TCRαβ clonal expansion status of T cells from B-ALL patients. **(B).** Summary of shared proportion of TCR clonotypes between T-cell subtypes. **(C).** Clonal expansion and transition ability comparison between CD4+ T-cell subsets of B-ALL patients. Kruskal–Wallis test. **(D).** Same as panel **(C)** but for CD8+ T-cell subsets. Kruskal–Wallis test. **(E).** Heatmap visualization of pairwise transition likelihood (based on clonotypes) of CD4+ T-cell subsets. **(F).** Same as panel **(E)** but for CD8+ T-cell subsets.

To analyze the clonal expansion status of T cells in B-ALL patients, we defined T cells with shared TCRαβ clonotype as “Clonal” and the other as “Non-clonal” or “Non-TCR detected” accordingly. We considered T cells with identical TCRαβ clonotypes differentiated from the same origin, thus, TCRαβ clonotype was used for T-cell lineage transition tracing. We observed that T-cell clonal expansion was mainly in Teff, Tem, Tex, and Trm-like cell clusters, and especially in Teff (**Figure 3A**). Then, we characterized the lineage transition by investigating the fraction of clonotypes shared between each pair of T-cell types (**Figure 3B**). The results revealed that between CD4+ T-cell subtypes, Teff cells had 14% clonotypes identical to Trm-like cells, and Trm-like cells had 13% clonotypes identical to Tex cells, which suggested CD4+ Teff, Trm-like, and Tex cells frequently share TCRαβ clonotypes. Similarly, in CD8+ T-cell subtypes, there were frequent overlaps of clonotypes between CD8+ Tem, Teff, Trm-like, and Tex cells: 13% clonotypes of Teff cells identical to Trm-like cells, 15% clonotypes of Tex cells identical to Trm-like cells, and 35% clonotypes of Teff cells identical to Tem cells. These findings suggested that those T-cell clusters with shared clonotypes may have the same origin. In addition, there were frequent overlaps of clonotypes between CD4+ and CD8+ Teff cells, which made us speculate that the CD4+ Teff cells were also involved in tumor killing (49). To confirm this, we quantified the clonal expansion (Expansion) and transition (Transition) index between T-cell subtypes (**Figures 3C, D**; *Methods*) (21). Among CD8+ T-cell subtypes, we found that Teff cells were of the most significant clonal expansion. Finally, we calculated the pairwise transition index (pIndex.tran) within CD4+ and CD8+ T-cell subsets (**Figures 3E, F** **)**. Among the CD4+ T-cell subtypes, Teff, Trm-like, and Tex cells had higher pairwise transition indices, with Trm-like and Tex cells having the highest pairwise transition (0.098) likelihood and Trm-like and Teff cells having less frequent pairwise transition (0.048) likelihood (**Figure 3E**). Similar results were also observed in the CD8+ subsets that Trm-like and Tex cells have the highest pairwise transition (0.16) likelihood (**Figure 3F**). Therefore, we deduced that there was a transition potential between Teff, Trm-like, and Tex cells, among which the transition potential is greater between Trm-like and Tex cells.

### Cell fate trajectory analysis confirms two transition directions of Trm-like cells

**Figure 4:**
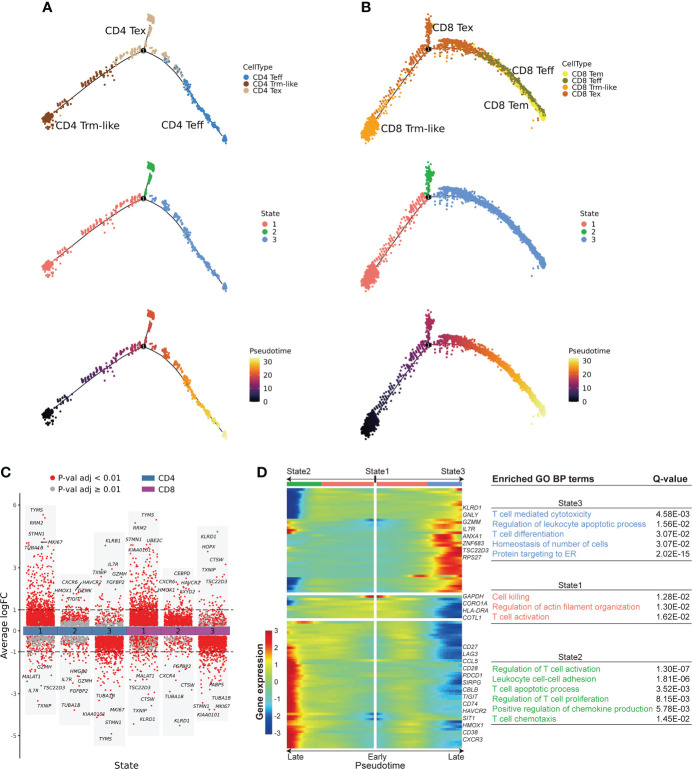
Cell fate trajectory of T cells from B-ALL patients. **(A).** The discriminative dimensionality reduction (DDR) tree visualization of selected subtypes of CD4+ T cell trajectory with cell type (top), state (middle), and pseudotime (bottom) information mapping, respectively. **(B).** Same as panel **(A)** but for selected CD8+ T-cell subsets. **(C).** Gene expression fold change between T cells in distinct cell fate trajectory states. The expression fold change of each gene in a T-cell state is determined by comparing to the average expression of the same gene in the other two states. **(D).** Left: expression heatmap of the top 100 genes that had the most significantly correlated (or anti-correlated) expression profile to the CD8+ T-cell fate pseudotime in panel **(B)** The correlation significances (Q values) were calculated by branched expression analysis modeling (BEAM). Right: enriched GO biological process terms for genes that were specifically expressed in CD8+ T cells of each state.

To further explore the transition relationships between those T-cell clusters with transition potentials (Teff, Trm-like, and Tex cells), we performed cell fate trajectory analysis for these CD4+ and CD8+ T-cell subsets. For the selected CD4+ T cells, the trajectories formed a branched tree shape with one splitting point, which divided the cells into three states. The three branched states (State1, State2, and State3) were significantly enriched with CD4+ Trm-like, Tex, and Teff cells, respectively (*P* < 0.001, *P* < 0.001, and *P* < 0.001, respectively; **Figure 4A**; **Supplementary Figure S5A**). We observed similar results of cell fate trajectory for the selected CD8+ T-cell subsets as well, with State1, State2, and State3 significantly enriched with CD8+ Trm-like, Tex, and Teff cells, respectively (*P* < 0.001, *P* < 0.001, and *P* < 0.001, respectively; **Figure 4B**; **Supplementary Figure S5B**). Furthermore, Tex cells from State2 branch had significantly higher exhaustion score (*P* < 0.001) than that from State1 and State3, which might suggest that Tex cells of State2 were terminally exhausted T cells (**Supplementary Figures S5C, D**). Similarly, we also found that Trm-like cells had the potential to differentiate into Teff in healthy individuals, despite that the number of Trm-like cells was low (**Supplementary Figure S5E**).

Next, we performed branched expression analysis modeling (BEAM; *Methods*) to discern the significant expression differences between the three branched states of the selected CD4+ and CD8+ T-cell subsets (**Figures 4C, D**; **Supplementary Figure S5F**). We selected the top 100 genes (**Supplementary Table S5**) that had the most significantly correlated (or anti-correlated) expression profile to the CD4+ and CD8+ T-cell fate trajectory pseudotime. We observed that the terminal portion of State2 significantly expressed cell exhaustion marker genes (42, 43) in both selected CD4+ and CD8+ T-cell subsets, while the terminal portion of State3 significantly expressed effector cell marker genes (**Figure 4D**; **Supplementary Figure S5F**). Furthermore, GO enrichment analysis of the pseudotime-aligned gene sets showed that T cells in State2 were enriched for genes related to “Lymphocyte apoptotic process” and “T cell apoptotic process,” and State3 was enriched for T-cell killing-related pathways, like “T cell mediated cytotoxicity,” and State1 was enriched for “Regulation of actin filament organization” and “T cell activation” (**Figure 4D**; **Supplementary Figure S5F**). According to these results, we speculate that Trm-like cells have the potential to be activated and become Teff cells but instead mainly transformed into an exhausted state in the leukemia microenvironment possibly due to binding of immunosuppressive checkpoint proteins (43, 45, 47, 50).

### Impact of Trm-like cell transformation direction on B-ALL prognosis

**Figure 5:**
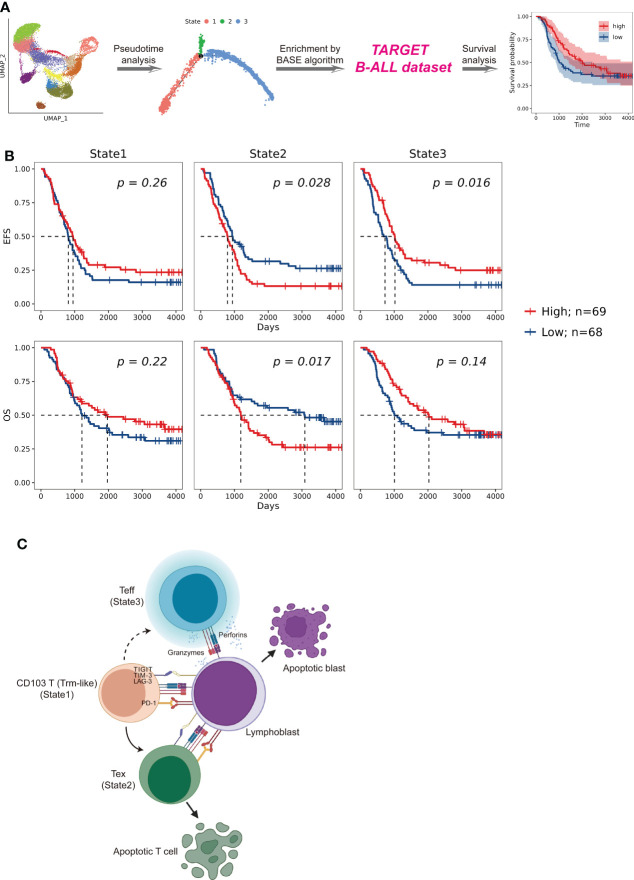
Patient survival impact comparison related to T cells in distinct cell fate trajectory states. **(A).** Schematic workflow of survival analysis based on the enrichment of CD4+ and CD8+ T cells in each state for patients enrolled in the TARGET (Therapeutically Applicable Research to Generate Effective Treatments) B-ALL database. The enrichment of T cells of each state in a patient was calculated by using BASE algorithm on the bulk-cell RNA-seq data from the corresponding individual. **(B).** Kaplan–Meier plots for the prognostic value of event-free survival (EFS) and overall survival (OS) according to the transcriptional signatures of each CD8+ T-cell state in patients from TARGET B-ALL dataset (n = 137). Patients were stratified into high and low level based on the median of the transcriptional signatures for each CD8+ T-cell state. *P* value was determined by log-rank test. **(C).** Proposed Trm-like cell functional transition model in B-ALL patient.

To investigate the potential prognostic significance of different transition fates of Trm-like cells in B-ALL patients, we summarized transcriptional signatures based on the DEGs in each branched state of the cell fate trajectory analysis from the selected CD4+ and CD8+ T-cell subsets for B-ALL survival analysis (**Figure 5A**; **Supplementary Table S6**; *Methods*). The results showed that patients with a high transcriptional signature of State2 of the selected CD8+ T-cell subset would have poor event-free survival (EFS) (*P* = 0.028) and OS (*P*  = 0.017), while patients with a high transcriptional signature of State3 of both the selected CD8+ and CD4+ T-cell subsets would have better EFS (*P*  =  0.016; *P*  = 0.005) (**Figure 5B**; **Supplementary Figure S5G**). These results of the survival analysis suggested that Trm-like cell (State1) transition toward Tex cells (State2) or Teff cells (State3) is potentially associated with poor or favorable prognosis of B-ALL patients, respectively (6, 47, 51).

## Discussion

Through the comprehensive single-cell transcriptome study of T cells on B-ALL, we speculate that in the leukemia microenvironment, circulating CD103+ T (Trm-like) cells are in a complex state that actively proliferate but are also prone to apoptosis and participate in the first-line immune function for leukemia cells. Upon recognizing lymphoblasts in the peripheral blood, Trm-like cells had the capability to transform into Teff cells for tumor killing; however, Trm-like cells tended to gradually become Tex cells possibly due to binding of immunosuppressive checkpoint proteins in the leukemia microenvironment (**Figure 5C**).

Trm cells are thought of as a third subset of memory T cells (52) and characterized by a distinct transcriptional program with CD103/CD69/CD49a as cell surface markers. It has been known for quite some time that Trm cells reside in the epithelium and occupy the frontline defense against antigens (13, 53). Trm cells usually provide strong long-term immune protection against antigen recurrence either by directly killing or by amplifying local recruitment of other innate and adaptive immune cells through the release of cytokines and chemokines (54). Recent studies have shown that Trm cells can rejoin the circulation pool and give rise to Teff cells in response to antigen stimulation (12, 13). In case of the leukemia environment, leukemia cells spread and express specific chemokines and cytokines as well as leukemia-associated antigens over the circulation system. Upon stimulation, Trm cells, which originally reside in tissues, reenter the circulation and allow for surveillance. It was reported that CD69, CCR7, and S1P1 likely played a role in modulating this migrating process (55). In our data of B-ALL, Trm-like cells were indeed elevated in peripheral blood and the low expression of CD69 was supposed to contribute to the migration.

Previous studies show that Trm and/or Trm-like cells appear to play an important role in the control of malignancies by tumor cell killing (56–59). A recent study showed that after the first-line treatment with immune checkpoint blockers in solid tumor patients, it was Trm and CD103+ cycling T cells that exert the powerful killing and antitumor effects in the tumor immune microenvironment (59). Notably, “Trm and/or Trm-like cell accumulation” was found in neoplasms of epithelial cell origin, such as uterine neck cancer (60), colorectal cancer (61), and lung cancer (57), as well as in non-epithelial cell origin, such as malignant glioma (62) and melanoma (63). The proportion of tumor-infiltrating T cells with a tissue-resident memory phenotype varied from 25% to 75%, which was partly due to the distinct specific markers used to identify these cells and the local tumor microenvironment. Thus, we speculate that Trm-like cells may be a distinct subcluster of tumor-infiltrating lymphocytes based on the expression of CD103. Our data further confirmed that Trm-like cells are of cytotoxic potential, expression of T-cell checkpoints, and capacity for cell proliferation. Tracking the dynamic shifts of these cells throughout the course of B-ALL progression would be of prognosis prediction value. In this way, Trm-like cells, as a part of the immune sensing network, monitor the fluctuations of the microenvironment and possibly play an important role in the occurrence and development of tumor or autoimmune diseases (47). Nevertheless, this study did not clarify from which tissue site these cells were derived and whether these cells could recirculate to tissue sites. Further understanding of the development, maintenance, and regulation of Trm-like cells would be crucial for addressing this question.

Through shared clonotype and cell fate trajectory analysis, we put forward a Trm-like cell evolution model in which Trm-like cells transform into Tex cells rather than Teff cells in the B-ALL microenvironment. We assumed that this might be due to the expression of specific cytokines in certain states (64). As the high transcriptional signature of State2 (Tex cells) links to a poor prognosis, while State3 (Teff cells) links to a favorable prognosis, the transition directions of Trm-like cells in B-ALL patients may be a potential prognostic factor for patient survival. Previous research had shown that the percentage of infiltration of Trm-like cells was positively correlated with a favorable prognosis. For example, the higher percentage of CD103+ tumor-infiltrating lymphocytes indicated a better prognosis of patients with high-grade serous ovarian cancer (65). Here, we also found that the transition fates of Trm-like cells were potentially related to patient prognosis. In addition, it has been reported that in solid tumors, programmed cell death protein 1 (PD-1) blocker treatment will induce Trm-like cells to transform toward Teff cells and improve the clinical outcome. As for immunoregulation, how to promote Trm-like cell transformation toward Teff cells will be of clinical value. Our study highlights the potential of Trm-like cells to increase our understanding of the leukemia microenvironment, potentially identifying new prognosis factors and guiding possible therapeutic strategies in the future.

Cancer immunotherapy has become the most promising treatment after surgery, radiotherapy, chemotherapy, and targeted therapy. How to achieve the proliferation and maintenance of tumor-specific CD103+ T cells may be critical to the clinical application of tumor immunotherapy. Despite being described in a number of solid tumor studies, the characteristics of Trm-like cells in B-ALL patients are revealed in this study. As a specific lymphocyte type in the leukemia microenvironment, the percentage of Trm-like cells in B-ALL was lower than TILs in solid tumor. However, considering the ease of sample acquisition, Trm-like cell responses in the peripheral blood may be an optimal indicator of treatment reactivity and long-term survival, at least in B-ALL. Further studies on the biological characteristics of Trm-like cells and their regulation on leukemia or solid tumors are urgently needed to explore novel immunotherapies.

## Conclusions

In conclusion, to the best of our knowledge, we uncovered a Trm-like cell subset with a high level of expression of cell proliferation- and exhaustion-related genes and with different expression profiles of T-cell migration-related genes in peripheral blood of B-ALL patients. Importantly, these Trm-like cells might have two opposite roles for the clinical outcome of B-ALL: transitioning into Teff cells leading to a favorable prognosis or into Tex cells resulting in a poor prognosis for B-ALL patients. Overall, these results provided unique insights on alterations of leukemia-related T cells and showed a possible immunotherapy direction and prognosis assessment model for B-ALL patients.

## Data availability statement

The original contributions presented in the study are included in the article/**Supplementary Material**. Further inquiries can be directed to the corresponding authors.

## Ethics statement

The studies involving human participants were reviewed and approved by Jinan University Ethics Committee. The patients/participants provided their written informed consent to participate in this study.

## Author contributions

WPL performed sequencing data analysis. WPL, XW, YLi and OL wrote the manuscript with input from YLi. XW interpreted the results. LL performed the flow cytometry and analyzed the data. LX helped with the flow cytometry. LM assisted with the data interpretation. JT, XZ, HZ, WL and GC provided the samples. YLi and OL conceived the study, designed the experiments, and oversaw the research project. All authors contributed to the article and approved the submitted version.

## Funding

This study was supported by the Natural Science Foundation of China (82070152 to YL, 81971301 and 32050410285 to OL, 81900110 to XW, 82000108 to LX), the Guangzhou Planned Project of Science and Technology (202002020039), the Initial Supporting Foundation of Jinan University (OL), the Fundamental Research Funds for the Central Universities (OL), and the research foundation of Guangzhou Women and Children’s Medical Center for Clinical Doctor (XW).

## Acknowledgments

We would like to thank Zhiyao Ren from Department of Systems Biomedical Sciences, School of Medicine, Jinan University, for technical support. We also thank Hui Zeng from Department of Hematology, First Affiliated Hospital, Jinan University, for providing samples for validation.

## Conflict of interest

The authors declare that the research was conducted in the absence of any commercial or financial relationships that could be construed as a potential conflict of interest.

The reviewer JL declared a shared parent affiliation with the author XW to the handling editor at the time of the review

## Publisher’s note

All claims expressed in this article are solely those of the authors and do not necessarily represent those of their affiliated organizations, or those of the publisher, the editors and the reviewers. Any product that may be evaluated in this article, or claim that may be made by its manufacturer, is not guaranteed or endorsed by the publisher.

## Supplementary material

Supplementary Figure 1 Characterization of peripheral immune cells from healthy individuals. **(A)**. UMAP visualization of PBMC single cell clusters from healthy individuals. Different clusters are depicted with distinct colors. The T cell cluster was further analyzed and compared to T cells from B-ALL patients. **(B)**. Dot plot of marker genes for each cell cluster in A. Color-scale indicates the mean of normalized expression of marker genes in each cell type, and dot size is proportional to the percentage of cells within each cell cluster expressing the marker genes. **(C)**. Expression heatmap of top 6 marker genes for each cell cluster. Maker genes for each cell type were ranked by expression fold-change between the corresponding cell type and the other cell types. Color-scale indicates the mean of normalized expression of genes in each cell type. **(D)**. Harmony and PCA visualization of T cells from B-ALL (top), healthy individuals (middle) and all combined (bottom). Different samples in each plot are depicted with distinct colors. **(E)**. UMAP visualization of T cell single cell clusters from B-ALL patients and healthy individuals when clustering was performed on all T cells from B-ALL patients and healthy individuals combined. T, T cell; B, B cell; NK, natural killer cell; Mono, monocyte; Ery, erythrocyte; DC, dendritic cell.

Supplementary Figure 2 Auxiliary annotation and marker genes expression of T cells. **(A)**. Characterization of the T cell clusters using independent reference gene signatures of ‘MonacoImmuneData’ dataset. Heatmap shows cross-labelling of T cell clusters of B-ALL patients defined in the present study (columns, reported as in ) versus reference gene signatures (rows) derived from the analyses in ‘MonacoImmuneData’ dataset, with color indicating log-transformed frequency. **(B)**. Same as A, but for healthy individuals. **(C)**. Projection of selected marker genes expression level on T cells from B-ALL patients. **(D)**. Same as C, but for healthy individuals. **(E)**. Expression heatmap of top 5 marker genes for each cell cluster from B-ALL and healthy. ITGAE is also included. Maker genes for each cell type were ranked by expression fold-change between the corresponding cell type and the other cell types. Color-scale indicates the mean of normalized expression of genes in each cell type. **(F)**. Boxplot comparison of proportion of exhausted T cells in the peripheral blood and among TILs in different solid tumor patients.

Supplementary Figure 3 Gating schemes used for the detection of CD103+ T cells. **(A)**. The gating strategies for CD103+CD3+, Ki-67+CD103+CD3+ and PD-1+CD103+CD3+ cells. **(B)**. The proportion of CD103+CD3+ T cells of B-ALL patients (n = 3) and healthy individuals (n = 3). Wilcoxon Rank Sum test (one-sided). **(C)**. The frequency of Ki-67+ (left) and PD-1+ (right) populations in CD103+CD3+ subset of B-ALL patients (n = 3) and healthy individuals (n = 3). Wilcoxon Rank Sum test (one-sided). BM, bone marrow; PB, peripheral blood.

Supplementary Figure 4 Pan-cancer leukocyte analysis by scRNA-seq. **(A)**. UMAP visualization of T and NK cell single cell clusters from pan-cancer. Different cell clusters are depicted with distinct colors. **(B)**. Dot plot of marker genes for each cell cluster. Color-scale indicates the mean of normalized expression of marker genes in each cluster, and dot size is proportional to the percentage of cells within each cell cluster expressing the marker genes. **(C)**. The ratio of cluster 19 (C19) to total T cells in different sites of multiple type of solid tumor patient. Kruskal-Wallis test. **P* < 0.05; ***P* < 0.01; ****P* < 0.001; NS not significant. BRC, breast cancer; CRC, colorectal cancer; HCC, hepatocellular carcinoma; NPC, nasopharyngeal carcinoma; NSCLC, non-small cell lung cancer; PDAC, pancreatic ductal adenocarcinoma; RCC, renal cell carcinoma; mRCC, metastatic RCC; Adj tissue, tissue adjacent to the tumor; Norm tissue, normal tissue.

Supplementary Figure 5 Ratio of cell type and exhaustion score of exhausted T cells in each state branch. **(A)**. Ratio of cell type in each state branch for CD4+ T cell subsets. **(B)**. Same as A, but for CD8+ T cell subsets. **(C)**. Exhaustion score of exhausted T cells in each state branch for CD4+ T cell subsets. **(D)**. Same as C, but for CD8+ T cell subsets. **(E)**. DDR tree visualization of selected subtypes of T cells trajectory from healthy with cell type (top) and pseudotime (bottom) information mapping, respectively. **(F)**. Left: expression heatmap of top 100 genes that had most significantly correlated (or anti-correlated) expression profile to the CD4+ T cell fate pseudotime in . The correlation significances (Q values) were calculated by branched expression analysis modeling (BEAM). Right: enriched GO biological process terms for genes that were specifically expressed in CD4+ T cells of each state. **(G)**. Kaplan-Meier plots for the prognostic value of event-free survival (EFS) and overall survival (OS) according to the transcriptional signatures of each CD4+ T cell state in patients from TARGET B-ALL dataset (n = 137). Patients were stratified into high and low level based on the median of the transcriptional signatures for each CD4+ T cell state. *P* value was determined by log-rank test. For boxplots, the outlines of the boxes represent the first and third quartiles. The line inside each box represents the median, and boundaries of the whiskers are found within the 1.5×IQR value. **P < 0.05; **P < 0.01; ***P < 0.001;* NS *not significant.* Chi-square test (two-sided) for A and B, Wilcoxon Rank Sum test (two-sided) for C and D.
