# Supplementary figures and images for: Single-cell profiling of T cells uncovers a tissue-resident memory-like T-cell subset associated with bidirectional prognosis for B-cell acute lymphoblastic leukemia
Source: Front Immunol. 2022 Dec 2;13:957436. doi: 10.3389/fimmu.2022.957436 (PMC9757161; doi:10.3389/fimmu.2022.957436)

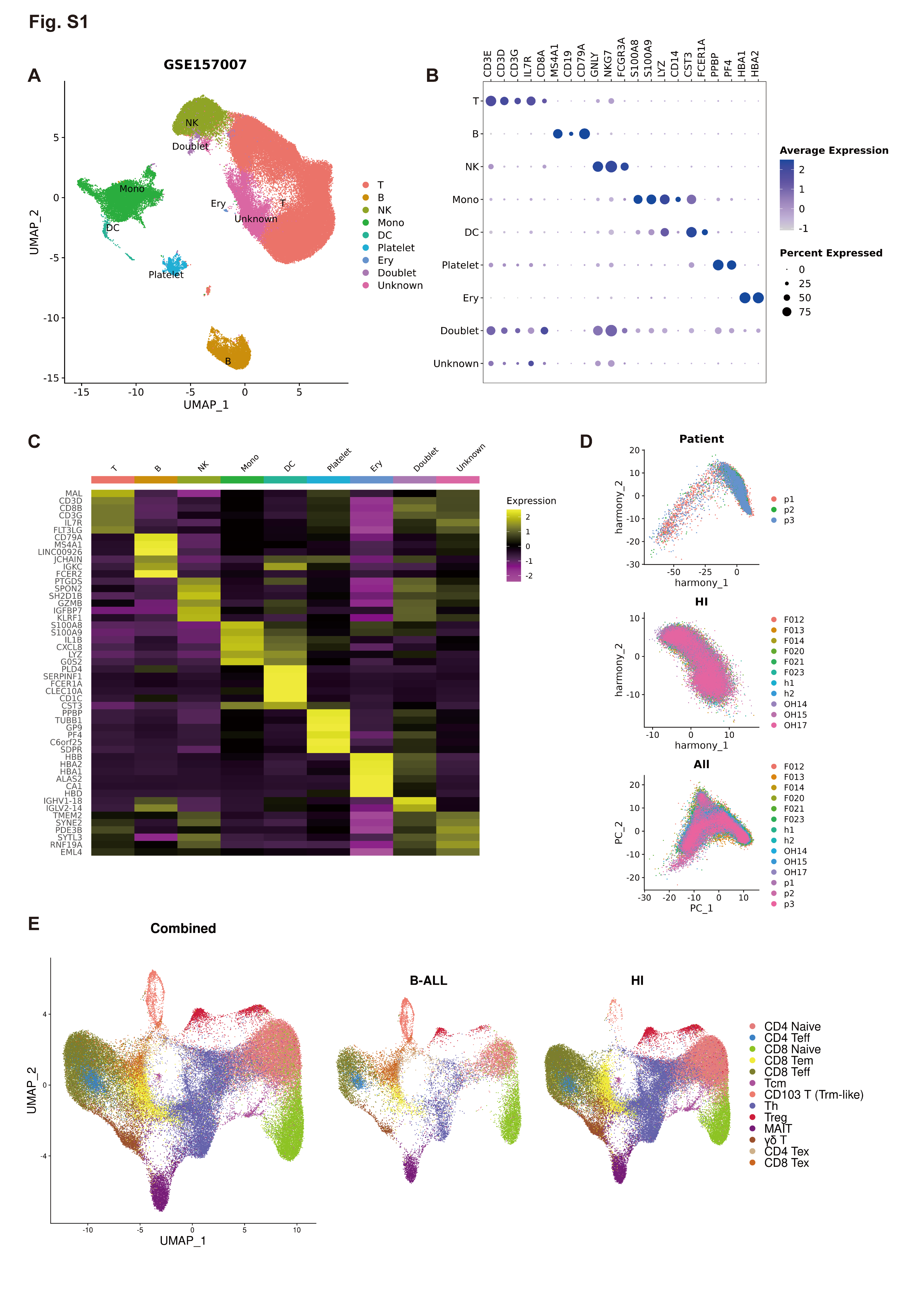

Supplement: Supplementary Figure 1 — Characterization of peripheral immune cells from healthy individuals. (A). UMAP visualization of PBMC single cell clusters from healthy individuals. Different clusters are depicted with distinct colors. The T cell cluster was further analyzed and compared to T cells from B-ALL patients. (B). Dot plot of marker genes for each cell cluster in A. Color-scale indicates the mean of normalized expression of marker genes in each cell type, and dot size is proportional to the percentage of cells within each cell cluster expressing the marker genes. (C). Expression heatmap of top 6 marker genes for each cell cluster. Maker genes for each cell type were ranked by expression fold-change between the corresponding cell type and the other cell types. Color-scale indicates the mean of normalized expression of genes in each cell type. (D). Harmony and PCA visualization of T cells from B-ALL (top), healthy individuals (middle) and all combined (bottom). Different samples in each plot are depicted with distinct colors. (E). UMAP visualization of T cell single cell clusters from B-ALL patients and healthy individuals when clustering was performed on all T cells from B-ALL patients and healthy individuals combined. T, T cell; B, B cell; NK, natural killer cell; Mono, monocyte; Ery, erythrocyte; DC, dendritic cell. [file Image_3.tif]

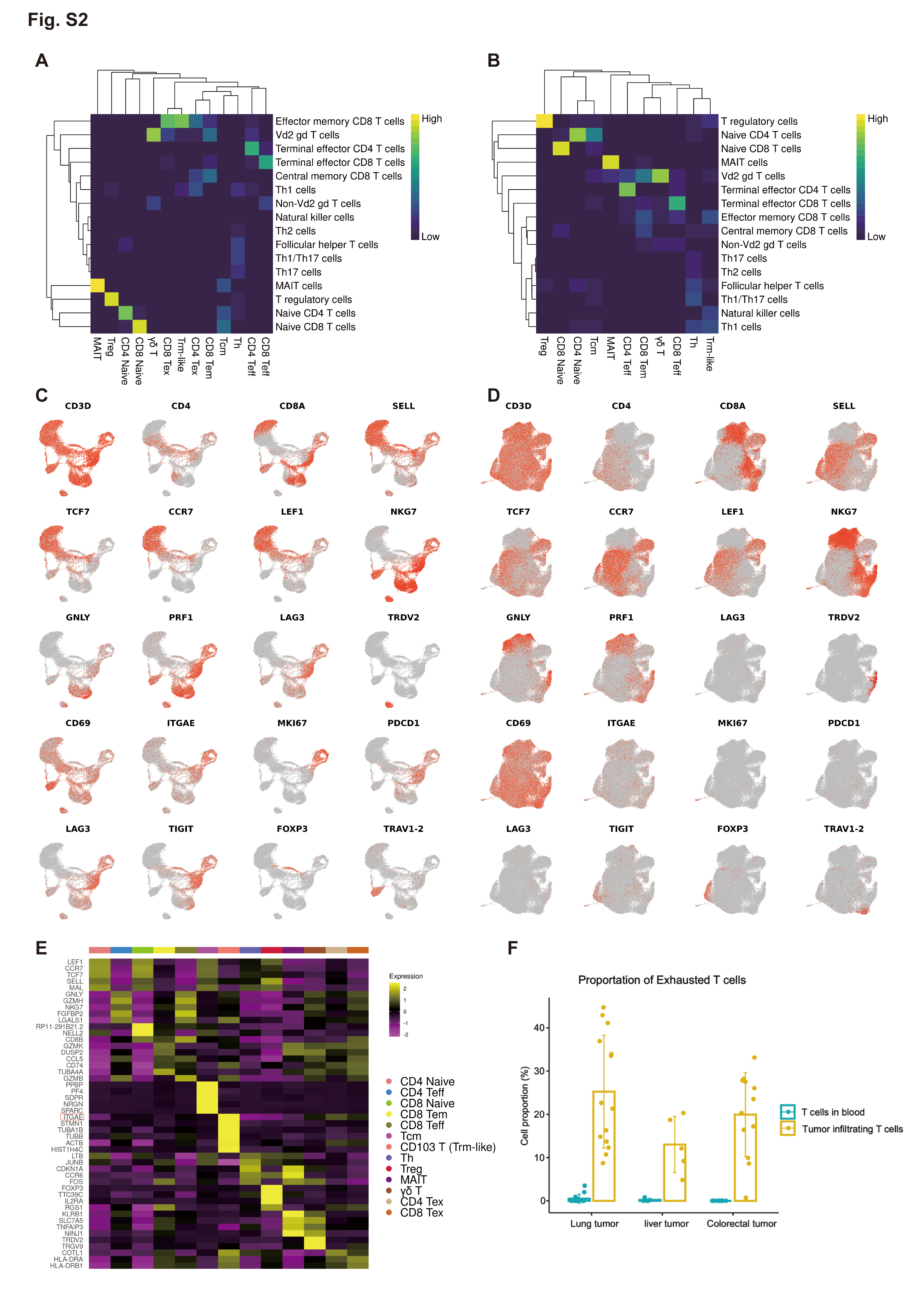

Supplement: Supplementary Figure 2 — Auxiliary annotation and marker genes expression of T cells. (A). Characterization of the T cell clusters using independent reference gene signatures of ‘MonacoImmuneData’ dataset. Heatmap shows cross-labelling of T cell clusters of B-ALL patients defined in the present study (columns, reported as in ) versus reference gene signatures (rows) derived from the analyses in ‘MonacoImmuneData’ dataset, with color indicating log-transformed frequency. (B). Same as A, but for healthy individuals. (C). Projection of selected marker genes expression level on T cells from B-ALL patients. (D). Same as C, but for healthy individuals. (E). Expression heatmap of top 5 marker genes for each cell cluster from B-ALL and healthy. ITGAE is also included. Maker genes for each cell type were ranked by expression fold-change between the corresponding cell type and the other cell types. Color-scale indicates the mean of normalized expression of genes in each cell type. (F). Boxplot comparison of proportion of exhausted T cells in the peripheral blood and among TILs in different solid tumor patients. [file Image_4.tif]

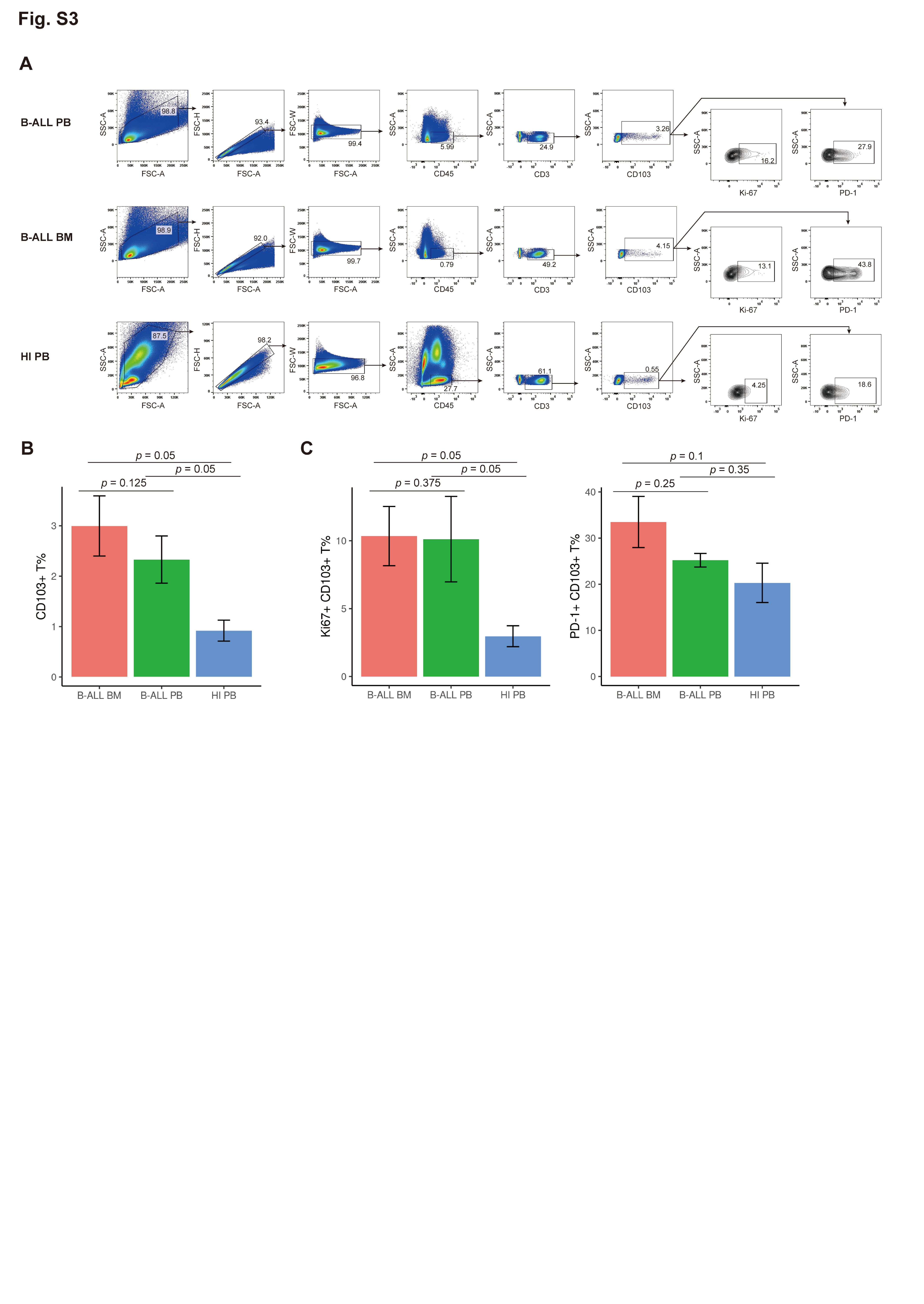

Supplement: Supplementary Figure 3 — Gating schemes used for the detection of CD103+ T cells. (A). The gating strategies for CD103+CD3+, Ki-67+CD103+CD3+ and PD-1+CD103+CD3+ cells. (B). The proportion of CD103+CD3+ T cells of B-ALL patients (n = 3) and healthy individuals (n = 3). Wilcoxon Rank Sum test (one-sided). (C). The frequency of Ki-67+ (left) and PD-1+ (right) populations in CD103+CD3+ subset of B-ALL patients (n = 3) and healthy individuals (n = 3). Wilcoxon Rank Sum test (one-sided). BM, bone marrow; PB, peripheral blood. [file Image_5.tif]

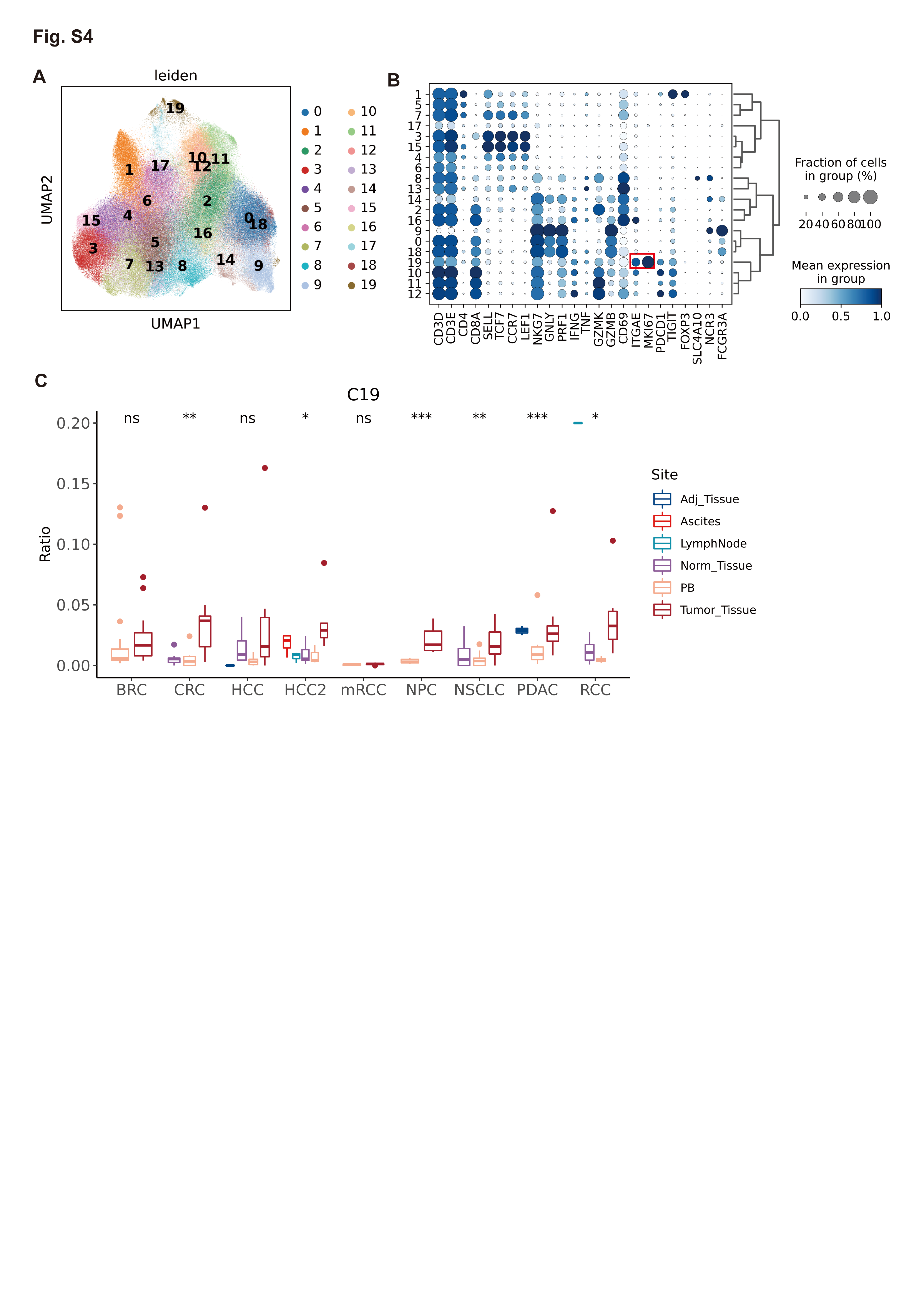

Supplement: Supplementary Figure 4 — Pan-cancer leukocyte analysis by scRNA-seq. (A). UMAP visualization of T and NK cell single cell clusters from pan-cancer. Different cell clusters are depicted with distinct colors. (B). Dot plot of marker genes for each cell cluster. Color-scale indicates the mean of normalized expression of marker genes in each cluster, and dot size is proportional to the percentage of cells within each cell cluster expressing the marker genes. (C). The ratio of cluster 19 (C19) to total T cells in different sites of multiple type of solid tumor patient. Kruskal-Wallis test. *P < 0.05; **P < 0.01; ***P < 0.001; NS not significant. BRC, breast cancer; CRC, colorectal cancer; HCC, hepatocellular carcinoma; NPC, nasopharyngeal carcinoma; NSCLC, non-small cell lung cancer; PDAC, pancreatic ductal adenocarcinoma; RCC, renal cell carcinoma; mRCC, metastatic RCC; Adj tissue, tissue adjacent to the tumor; Norm tissue, normal tissue. [file Image_6.tif]

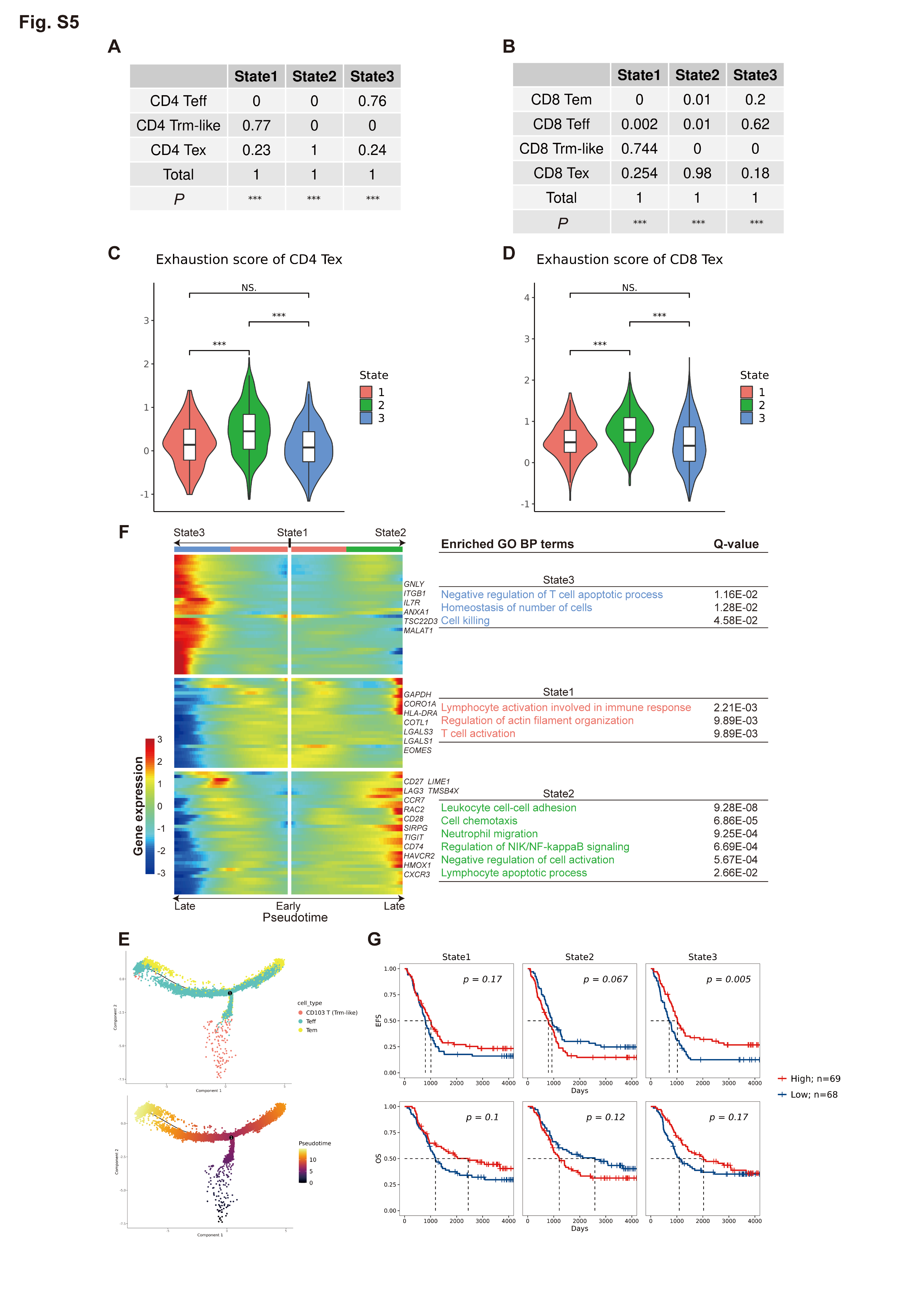

Supplement: Supplementary Figure 5 — Ratio of cell type and exhaustion score of exhausted T cells in each state branch. (A). Ratio of cell type in each state branch for CD4+ T cell subsets. (B). Same as A, but for CD8+ T cell subsets. (C). Exhaustion score of exhausted T cells in each state branch for CD4+ T cell subsets. (D). Same as C, but for CD8+ T cell subsets. (E). DDR tree visualization of selected subtypes of T cells trajectory from healthy with cell type (top) and pseudotime (bottom) information mapping, respectively. (F). Left: expression heatmap of top 100 genes that had most significantly correlated (or anti-correlated) expression profile to the CD4+ T cell fate pseudotime in . The correlation significances (Q values) were calculated by branched expression analysis modeling (BEAM). Right: enriched GO biological process terms for genes that were specifically expressed in CD4+ T cells of each state. (G). Kaplan-Meier plots for the prognostic value of event-free survival (EFS) and overall survival (OS) according to the transcriptional signatures of each CD4+ T cell state in patients from TARGET B-ALL dataset (n = 137). Patients were stratified into high and low level based on the median of the transcriptional signatures for each CD4+ T cell state. P value was determined by log-rank test. For boxplots, the outlines of the boxes represent the first and third quartiles. The line inside each box represents the median, and boundaries of the whiskers are found within the 1.5×IQR value. *P < 0.05; **P < 0.01; ***P < 0.001; NS not significant. Chi-square test (two-sided) for A and B, Wilcoxon Rank Sum test (two-sided) for C and D. [file Image_7.tif]

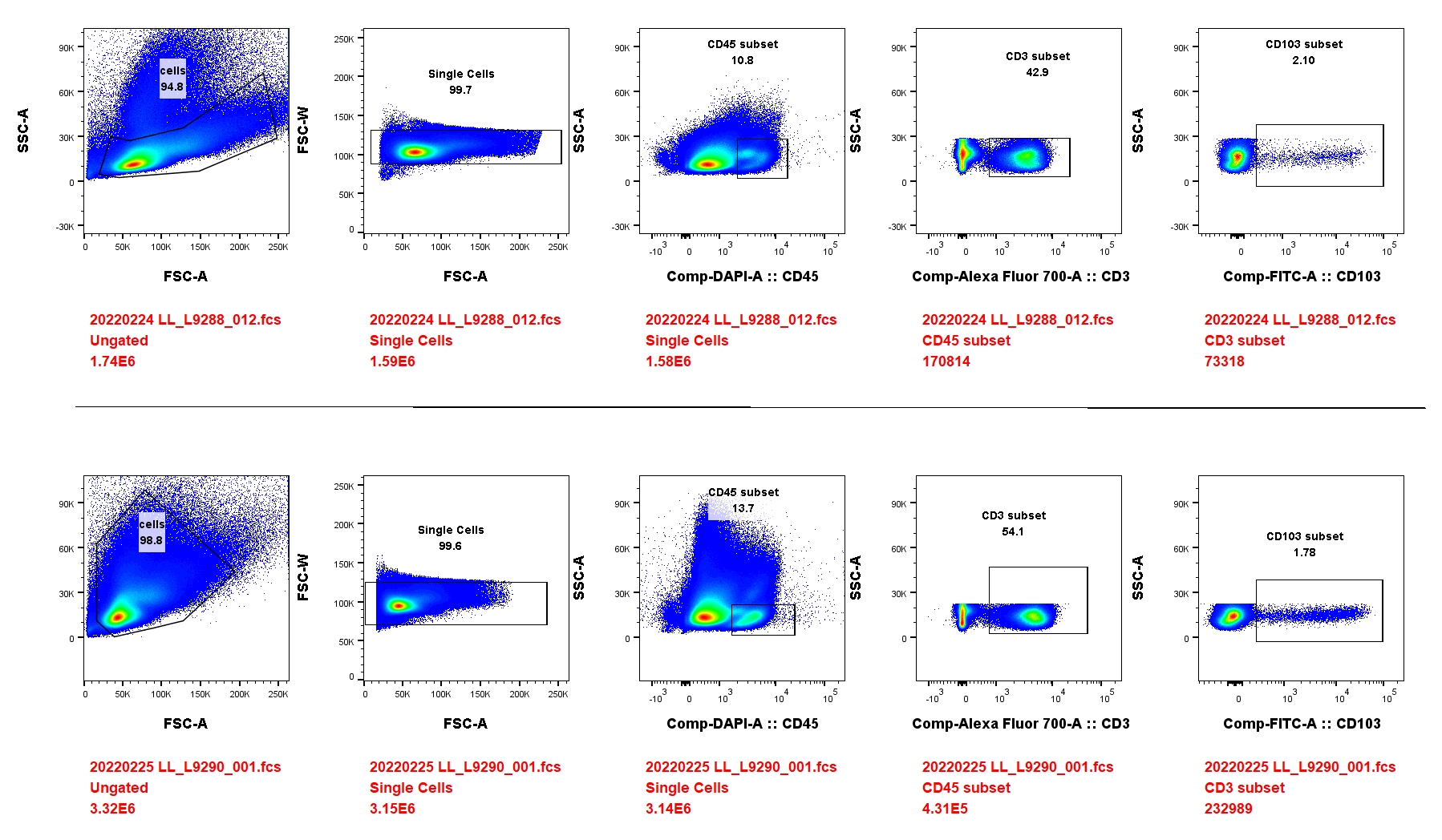

Supplement: Supplementary file 6 [file Image_1.jpeg]

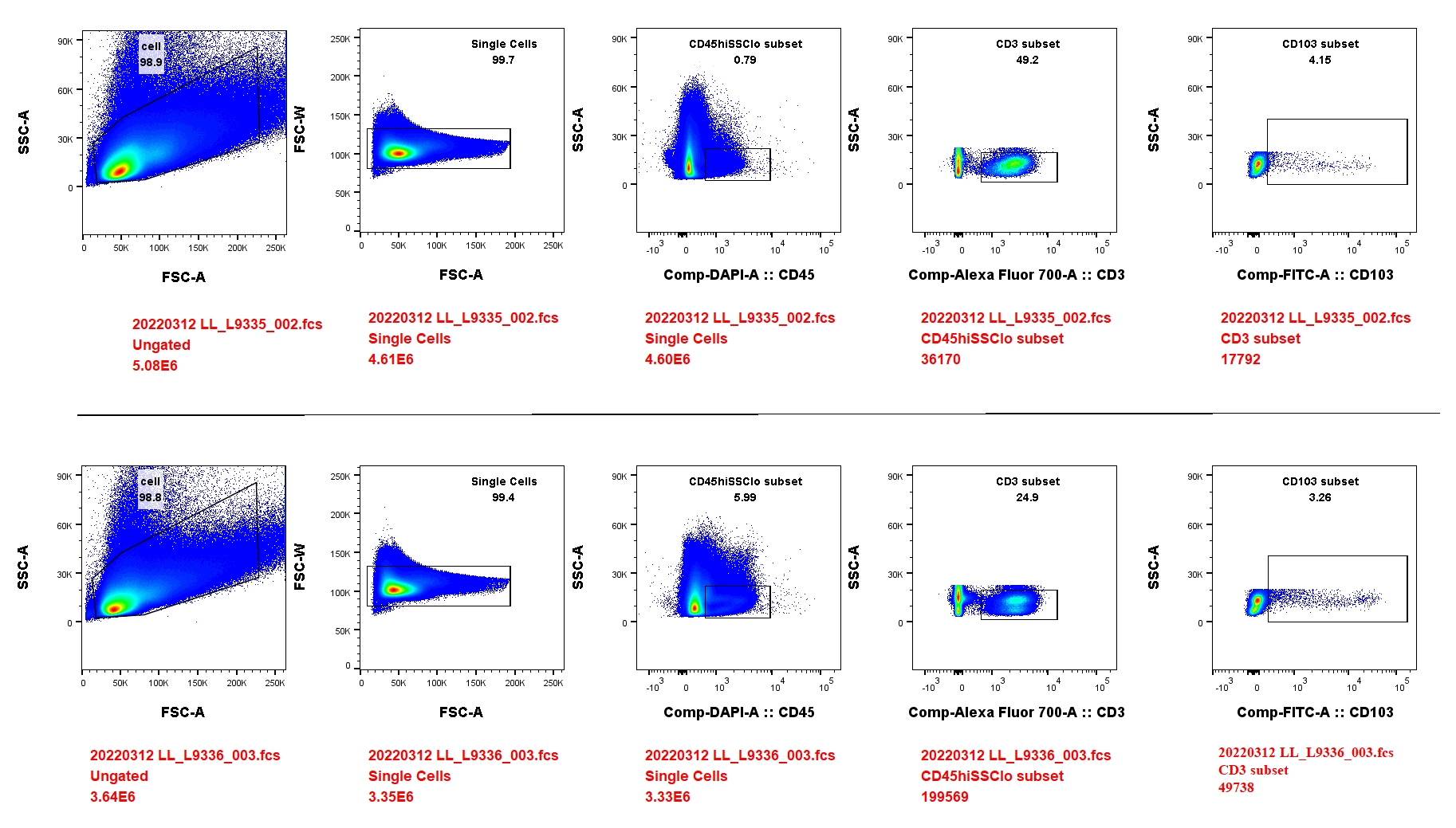

Supplement: Supplementary file 7 [file Image_2.jpeg]
