# Supplementary material for: Single-cell profiling of T cells uncovers a tissue-resident memory-like T-cell subset associated with bidirectional prognosis for B-cell acute lymphoblastic leukemia
Source: Front Immunol. 2022 Dec 2;13:957436. doi: 10.3389/fimmu.2022.957436 (PMC9757161; doi:10.3389/fimmu.2022.957436)
Supplement: Supplementary file 8 [file DataSheet_1.docx]

**List of supplementary material**

1. **Supplementary Tables**
   1. Supplementary Table 1. Sample information of the scRNA-Seq data used in this study.
   2. Supplementary Table 2. List of genes for functional modules
   3. Supplementary Table 3. Enriched GO biological processes of the up-regulated genes in B-ALL Trm cells in comparison to HI (*P* value < 0.05)
   4. Supplementary Table 4. Enriched KEGG signaling pathway of the up-regulated genes in B-ALL Trm cells in comparison to HI (*P* value < 0.05)
   5. Supplementary Table 5. Top 100 genes that had most significantly correlated (or anti-correlated) expression profile (ordered by Q value) to the CD4+ and CD8+ T cell fate trajectory pseudotime
   6. Supplementary Table 6. DEGs among different branched state of cell fate trajectory analysis (adjusted *P* value < 0.01 & absolute value of average logaritmic fold change ≥ 1)
   7. Supplementary Table 7. Sample information for flow cytometry cohort
2. **Flow cytometry (https://www.jianguoyun.com/c/sd/1547602/7e05f3a34b8d5d4f)**
   1. Raw data of flow cytometry
      1. 20220224 LL_FMO L9288_011.fcs
      2. 20220224 LL_L9288_012.fcs
      3. 20220225 LL_L9290_001.fcs
      4. 20220312 LL_FMO_001.fcs
      5. 20220312 LL_L9335_002.fcs
      6. 20220312 LL_L9336_003.fcs
      7. 20220322 LL_HI 1_001.fcs
      8. 20220322 LL_HI 2_002.fcs
      9. 20221005HB_FMO_001.fcs
      10. 20221005HB_PB_002.fcs
      11. 20221005HB_BM_003.fcs
   2. Initial image of flow cytometry
      1. flow cytometry_9288_9290.jpg
      2. flow cytometry_9335_9336.jpg
3. **GEO dataset（Supplementary Table 1）**
   1. GSE172158 (<https://www.ncbi.nlm.nih.gov/geo/query/acc.cgi?acc=GSE172158>)
   2. GSE157007 (<https://www.ncbi.nlm.nih.gov/geo/query/acc.cgi?acc=GSE157007>)
4. **R script**
   1. R script.docx (https://www.jianguoyun.com/c/sd/1547602/7e05f3a34b8d5d4f)
