# Supplementary material for: Single-cell profiling of T cells uncovers a tissue-resident memory-like T-cell subset associated with bidirectional prognosis for B-cell acute lymphoblastic leukemia
Source: Front Immunol. 2022 Dec 2;13:957436. doi: 10.3389/fimmu.2022.957436 (PMC9757161; doi:10.3389/fimmu.2022.957436)
Supplement: Supplementary file 9 [file DataSheet_2.docx]

library(dplyr)

library(Seurat)

library(patchwork)

library(harmony)

library(tidyverse)

library(monocle)

library(ggsignif)

library(patchwork)

library(cluster)

library(pheatmap)

library(RColorBrewer)

library(Startrac)

library(ggrepel)

library(reshape2)

library(gridExtra)

library(grid)

library(survival)

library(survminer)

library(viridis)

library(clusterProfiler)

############Figure1#################

load("B-ALL.Rdata")

load("Healthy.Rdata")

###########A

data<- FetchData(merges,vars = c("UMAP_1","UMAP_2","CellType"))

levels(data$CellType)

data$CellType <- as.character(data$CellType)

data$CellType[data$CellType=="CD4 Naive"] <- "1: CD4 Naive"

data$CellType[data$CellType=="CD4 Tff"] <- "2: CD4 Tff"

data$CellType[data$CellType=="CD8 Naive"] <- "3: CD8 Naive"

data$CellType[data$CellType=="CD8 Tem"] <- "4: CD8 Tem"

data$CellType[data$CellType=="CD8 Tff"] <- "5: CD8 Tff"

data$CellType[data$CellType=="Tcm"] <- "6: Tcm"

data$CellType[data$CellType=="Trm"] <- "7: Trm"

data$CellType[data$CellType=="Th"] <- "8: Th"

data$CellType[data$CellType=="Treg"] <- "9: Treg"

data$CellType[data$CellType=="MAIT"] <- "10: MAIT"

data$CellType[data$CellType=="γδ T"] <- "11: γδ T"

data$CellType[data$CellType=="CD4 Tex"] <- "12: CD4 Tex"

data$CellType[data$CellType=="CD8 Tex"] <- "13: CD8 Tex"

data$CellType <- factor(data$CellType,levels = c("1: CD4 Naive","2: CD4 Tff","3: CD8 Naive","4: CD8 Tem","5: CD8 Tff",

"6: Tcm","7: Trm","8: Th","9: Treg","10: MAIT","11: γδ T","12: CD4 Tex","13: CD8 Tex"))

cell_type_med <- data %>%

group_by(CellType) %>%

summarise(

UMAP_1 = median(UMAP_1),

UMAP_2 = median(UMAP_2)

)

cell_type_med$ident <- c(1:11)

Theme2<-theme(title = element_text(size = 14,face = "bold.italic"),panel.background = element_blank(),panel.border = element_blank(),panel.grid=element_blank(), axis.title = element_text(color='black',size=16),axis.ticks.length = unit(0.4,"lines"),axis.ticks = element_blank(),axis.line = element_blank(),axis.text=element_blank(),legend.title = element_text(size = 14,face = "plain"),legend.text=element_text(size=14),legend.key=element_blank(),legend.key.height=unit(0.5,'cm'),

legend.position = "left",aspect.ratio = 1,plot.title = element_text(hjust = 0.5))

p1.1<-ggplot(data,aes(x=UMAP_1,y=UMAP_2))+geom_point(aes(color=CellType),size=0.5)+scale_color_manual(values = c("#F08080","#1E90FF","#7CFC00","#FFFF00",

"#808000","#FF00FF","#FA8072","#7B68EE","#DC143C","#800080","#A0522D","#D2B48C","#D2691E","#87CEEB","#40E0D0"))+geom_label(aes(label=ident),cell_type_med,nudge_x=0,alpha=0,size=6,label.size = NA)+labs(x=" ",y=" ")+theme_bw()+Theme2+

guides(colour = guide_legend(override.aes = list(size=3.5),ncol = 2))+NoLegend()+ggtitle("HI")

data<- FetchData(scRNA1,vars = c("UMAP_1","UMAP_2","CellType"))

levels(data$CellType)

data$CellType <- as.character(data$CellType)

data$CellType[data$CellType=="CD4 Naive"] <- "1: CD4 Naive"

data$CellType[data$CellType=="CD4 Tff"] <- "2: CD4 Tff"

data$CellType[data$CellType=="CD8 Naive"] <- "3: CD8 Naive"

data$CellType[data$CellType=="CD8 Tem"] <- "4: CD8 Tem"

data$CellType[data$CellType=="CD8 Tff"] <- "5: CD8 Tff"

data$CellType[data$CellType=="Tcm"] <- "6: Tcm"

data$CellType[data$CellType=="Trm"] <- "7: Trm"

data$CellType[data$CellType=="Th"] <- "8: Th"

data$CellType[data$CellType=="Treg"] <- "9: Treg"

data$CellType[data$CellType=="MAIT"] <- "10: MAIT"

data$CellType[data$CellType=="γδ T"] <- "11: γδ T"

data$CellType[data$CellType=="CD4 Tex"] <- "12: CD4 Tex"

data$CellType[data$CellType=="CD8 Tex"] <- "13: CD8 Tex"

data$CellType <- factor(data$CellType,levels = c("1: CD4 Naive","2: CD4 Tff","3: CD8 Naive","4: CD8 Tem","5: CD8 Tff",

"6: Tcm","7: Trm","8: Th","9: Treg","10: MAIT","11: γδ T","12: CD4 Tex","13: CD8 Tex"))

cell_type_med <- data %>%

group_by(CellType) %>%

summarise(

UMAP_1 = median(UMAP_1),

UMAP_2 = median(UMAP_2)

)

cell_type_med$ident <- c(1:13)

p1<-ggplot(data,aes(x=UMAP_1,y=UMAP_2))+geom_point(aes(color=CellType),size=0.5)+scale_color_manual(values = c("#F08080","#1E90FF","#7CFC00","#FFFF00",

"#808000","#FF00FF","#FA8072","#7B68EE","#DC143C","#800080","#A0522D","#D2B48C","#D2691E","#87CEEB","#40E0D0"))+geom_label(aes(label=ident),cell_type_med,nudge_x=0,alpha=0,size=6,label.size = NA)+labs(x=" ",y=" ")+theme_bw()+Theme2+

guides(colour = guide_legend(override.aes = list(size=3.5),ncol = 1))+geom_segment(aes(x = min(data$UMAP_1) , y = min(data$UMAP_2),xend = min(data$UMAP_1) +3.5, yend = min(data$UMAP_2)),colour = "black", size=1,arrow = arrow(length = unit(0.3,"cm")))+ geom_segment(aes(x = min(data$UMAP_1),y = min(data$UMAP_2),xend = min(data$UMAP_1),yend = min(data$UMAP_2) + 3.5),colour = "black", size=1,arrow = arrow(length = unit(0.3,"cm")))+

annotate("text", x = min(data$UMAP_1) +2, y = min(data$UMAP_2) -1, label = "UMAP_1",color="black",size = 4, fontface="bold" ) +annotate("text", x = min(data$UMAP_1) -1, y = min(data$UMAP_2) + 2, label = "UMAP_2",color="black",size = 4, fontface="bold" ,angle=90)+ggtitle("B-ALL")

p1+p1.1

ggsave(plot = p1+p1.1,filename = "Figure1_A.png",width =11,height = 6,dpi = 1000)

###########B

scRNA1$CellType <- factor(scRNA1$CellType,levels = c(c("CD4 Naive","CD8 Naive","Tcm","CD8 Tem","Trm","CD4 Tff","CD8 Tff","γδ T","MAIT","Th","Treg","CD4 Tex","CD8 Tex")))

merges$CellType <- factor(merges$CellType,levels = c(c("CD4 Naive","CD8 Naive","Tcm","CD8 Tem","Trm","CD4 Tff","CD8 Tff","CD4 Tex","CD8 Tex","γδ T","MAIT","Th","Treg")))

p2<- DotPlot(scRNA1,scale = T,col.min = 0,col.max = 3,assay = "RNA" ,scale.max = 100,group.by = "CellType",#cols = c("grey","red"),#split.by = "group",cols = c("red","blue","green"),

features = c("CD3D","CD3E","CD4","CD8A","SELL","TCF7","CCR7","LEF1","NKG7","GNLY","PRF1","IFNG","GZMK","GZMB","CD69","ITGAE","MKI67","PDCD1","HAVCR2","LAG3","CTLA4","TIGIT","FOXP3","TRDC","TRDV2","TRAV1-2")) +theme_test()+ggtitle("B-ALL")+

xlab("Others\nmarkers\nExhaustion\nmarkers\nTrm\nmarkers\nEffector\ncytokines\nNaïve\nmakers\nLineage\nmarkers")+ylab("")+

coord_flip()+theme(axis.text.x = element_text(angle = 45, vjust =1.0, hjust=1.0,colour = "black"),axis.text.y = element_text(face = "italic",colour = "black"),

plot.title = element_text(hjust = 0.5,vjust = 0,face = "bold",size = 15),axis.title.y = element_text(size = 9,hjust = 0.5,vjust = 0,angle = 0),

text=element_text(size=14,colour = "black"),legend.title=element_text(size=11),legend.text=element_text(size=11))+scale_color_gradientn(values = seq(0,1,0.1),colours = c("grey","blue"))+NoLegend()

p2.1 <- DotPlot(merges,scale = T,col.min = 0,col.max = 3,assay = "RNA" ,scale.max = 100,group.by = "CellType",#cols = c("grey","red"),#split.by = "group",cols = c("red","blue","green"),

features = c("CD3D","CD3E","CD4","CD8A","SELL","TCF7","CCR7","LEF1","NKG7","GNLY","PRF1","IFNG","GZMK","GZMB","CD69","ITGAE","MKI67","PDCD1","HAVCR2","LAG3","CTLA4","TIGIT","FOXP3","TRDC","TRDV2","TRAV1-2"))+theme_test() + ggtitle("HI")+xlab("")+ylab("")+

coord_flip()+theme(axis.text.y = element_blank(),axis.ticks.y = element_blank(),axis.text.x = element_text(angle = 45, vjust =1.0, hjust=1.0,colour = "black"),text=element_text(size=14,colour = "black"),legend.title=element_text(size=10),legend.text=element_text(size=10),

plot.title = element_text(hjust = 0.5,vjust = 0,face = "bold",size = 15))+scale_color_gradientn(values = seq(0,1,0.1),colours = c("grey","blue"))

p2+p2.1

ggsave(plot = p2+p2.1,filename = "Figure1_B.png",width = 10 ,height = 6,dpi = 1000)

###########C and D

scRNA1$Group <- "B-ALL"

merges$Group <- "HI"

m3 <- data.frame(prop.table(table(scRNA1$batch,scRNA1$CellType),margin = 1))

m3$Group <- "B-ALL"

m4 <- data.frame(prop.table(table(merges$batch,merges$CellType),margin = 1))

m4$Group <- "HI"

m5 <- rbind(m3,m4)

colnames(m5)[1:3] <- c("batch","CellType","ratio")

m5 <- subset(m5,CellType=="Trm")

m5$batch <- factor(m5$batch,levels = c("p1","p2","p3","h1","h2","F012","F013","F014","F020","F021","F023","OH14","OH15","OH17"))

m5$ratio <- round(m5$ratio*100,2)

p4 <- ggplot(m5, aes(x=batch, y=ratio,group=CellType)) +ylim(0,15)+

geom_line() +geom_point(aes(color=Group),size=2)+

geom_text(data = m5,aes(x=batch,y=ratio,label = ratio,colour=Group),size=4,nudge_x = 0.20,nudge_y = 0.5)+

theme_bw()+ylab("Proportion of Trm(%)")+

theme(text = element_text(size=16,colour = "black"),axis.text.y = element_text(size = 14,color = "black"),axis.text.x = element_text(size = 14,color = "black",angle = 45, vjust =1.0, hjust=1.0),axis.title.x = element_blank(),

legend.title = element_blank(),legend.text = element_text(size=16))+ guides(color=guide_legend(override.aes = list(size=4)))

m1 <- data.frame(prop.table(table(scRNA1$Group,scRNA1$CellType)))

m2 <- data.frame(prop.table(table(merges$Group,merges$CellType)))

m <- rbind(m1,m2)

m$Freq <- m$Freq*100

colnames(m) <- c("Group","CellType","Proportion(%)")

m$x <- rep(1:2, each=13)

p3<- ggplot(m, aes(x, `Proportion(%)`, fill=CellType)) + geom_area(size=.5, colour="white") + scale_fill_manual(values=c("#F08080","#1E90FF","#7CFC00","#FFFF00",

"#808000","#FF00FF","#FA8072","#7B68EE","#DC143C","#800080","#A0522D","#D2B48C","#D2691E","#87CEEB","#40E0D0")) +

theme_bw() +scale_x_continuous(breaks=seq(1, 2, 1))+xlab("B-ALL HI")+

theme(text = element_text(size=16,colour = "black"),axis.text.y = element_text(size = 14,color = "black"),

axis.text.x = element_blank(),axis.title.x = element_text(size = 14,color = "black",hjust = 0.5,vjust =0),

legend.title = element_blank())

p3+p4+plot_layout(nrow = 1,widths = c(1,4))

ggsave(p3+p4+plot_layout(nrow = 1,widths = c(1,3)),filename = "Figure1_C-D.png.png",width = 13,height = 6,dpi = 1000)

############Figure2#################

load("Trm.Rdata")

###########A

compaired <- list(c("B-ALL","HI"))

data <- FetchData(Trm,vars = c("Group","Proliferation_score1"))

p7.1 <- ggplot(data,aes(x=Group,y=Proliferation_score1,fill=Group))+geom_violin()+theme_classic()+ggtitle("Proliferation score")+scale_x_discrete("")+

theme(title = element_text(face = "plain",size = 12),

legend.title = element_blank(),

legend.text = element_text(size = 12),

legend.key.size = unit(0.5, 'cm'), #change legend key size

aspect.ratio = 1.2,

axis.text.x = element_blank(),

axis.text.y = element_text(size = 10),

axis.title.x = element_blank(),

axis.title.y = element_blank(),

panel.border = element_blank(),

axis.ticks.x = element_blank(),

plot.title=element_text(hjust = 0.5,vjust = 5))+

geom_boxplot(width=0.2,position=position_dodge(0.9),outlier.colour = NA,fill="white")+

geom_signif(comparisons = compaired,

step_increase = 0.2,

map_signif_level = T,

test = wilcox.test,textsize = 3.5)+NoLegend()

data <- FetchData(Trm,vars = c("Group","Cytotoxicity_score1"))

p7.2 <- ggplot(data,aes(x=Group,y=Cytotoxicity_score1,fill=Group))+geom_violin()+theme_classic()+ggtitle("Cytotoxicity score")+scale_x_discrete("")+

theme(title = element_text(face = "plain",size = 12),

legend.title = element_blank(),

legend.text = element_text(size = 12),

legend.key.size = unit(0.5, 'cm'), #change legend key size

aspect.ratio = 1.2,

axis.text.x = element_blank(),

axis.text.y = element_text(size = 10),

axis.title.x = element_blank(),

axis.title.y = element_blank(),

panel.border = element_blank(),

axis.ticks.x = element_blank(),

plot.title=element_text(hjust = 0.5,vjust = 5))+

geom_boxplot(width=0.2,position=position_dodge(0.9),outlier.colour = NA,fill="white")+

geom_signif(comparisons = compaired,

step_increase = 0.2,

map_signif_level = T,

test = wilcox.test,textsize = 3.5)+NoLegend()

data <- FetchData(Trm,vars = c("Group","Exhausted_score1"))

p7.3 <- ggplot(data,aes(x=Group,y=Exhausted_score1,fill=Group))+geom_violin()+theme_classic()+ggtitle("Exhausted score")+scale_x_discrete("")+

theme(title = element_text(face = "plain",size = 12),

legend.title = element_blank(),

legend.text = element_text(size = 12),

legend.key.size = unit(0.5, 'cm'), #change legend key size

aspect.ratio = 1.2,

axis.text.x = element_blank(),

axis.text.y = element_text(size = 10),

axis.title.x = element_blank(),

axis.title.y = element_blank(),

panel.border = element_blank(),

axis.ticks.x = element_blank(),

plot.title=element_text(hjust = 0.5,vjust = 5))+

geom_boxplot(width=0.2,position=position_dodge(0.9),outlier.colour = NA,fill="white")+

geom_signif(comparisons = compaired,

step_increase = 0.2,

map_signif_level = T,

test = wilcox.test,textsize = 3.5)+NoLegend()

data <- FetchData(Trm,vars = c("Group","Tissue_residuce_score1"))

p7.4 <- ggplot(data,aes(x=Group,y=Tissue_residuce_score1,fill=Group))+geom_violin()+theme_classic()+ggtitle("Tissue-resident score")+scale_x_discrete("")+

theme(title = element_text(face = "plain",size = 12),

legend.title = element_blank(),

legend.text = element_text(size = 12),

legend.key.size = unit(0.5, 'cm'), #change legend key size

aspect.ratio = 1.2,

axis.text.x = element_blank(),

axis.text.y = element_text(size = 10),

axis.title.x = element_blank(),

axis.title.y = element_blank(),

panel.border = element_blank(),

axis.ticks.x = element_blank(),

plot.title=element_text(hjust = 0.5,vjust = 5) )+

geom_boxplot(width=0.2,position=position_dodge(0.9),outlier.colour = NA,fill="white")+

geom_signif(comparisons = compaired,

step_increase = 0.2,

map_signif_level = T,

test = wilcox.test,textsize = 3.5)

p7.1+p7.2+p7.3+p7.4+plot_layout(nrow = 1)

ggsave(plot = p7.1+p7.2+p7.3+p7.4+plot_layout(nrow = 1),filename = "Figure2_A.png",width = 13,height = 5,dpi = 1000)

###########B

p10.1 <- ggplot(data = GO_select,mapping = aes(x=Description,y=Count,fill=`-Log10(P)`))+geom_bar(stat = "identity")+coord_flip()+theme_classic()+scale_fill_gradient(low = "thistle",high = "darkviolet")+

ggtitle("GO biological process")+

theme(title = element_text(size = 6,colour = "black"),axis.title = element_blank(),axis.text = element_text(size = 6,colour = "black"),axis.line = element_line(size = 0.2),axis.ticks = element_line(size = 0.2),

legend.text = element_text(size = 6,colour = "black"),legend.title = element_text(size = 6,colour = "black"),legend.key.height = unit(0.3,"cm"),legend.key.width = unit(0.25,"cm"))

p10.2 <- ggplot(data = kegg_select,mapping = aes(x=Description,y=Count,fill=`-Log10(P)`))+geom_bar(stat = "identity")+coord_flip()+theme_classic()+scale_fill_gradient(low = "mistyrose",high = "darkred")+

ggtitle("KEGG signaling pathway")+

theme(title = element_text(size = 6,colour = "black"),axis.title = element_blank(),axis.text = element_text(size = 6,colour = "black"),axis.line = element_line(size = 0.2),axis.ticks = element_line(size = 0.2),

legend.text = element_text(size = 6,colour = "black"),legend.title = element_text(size = 6,colour = "black"),legend.key.height = unit(0.3,"cm"),legend.key.width = unit(0.25,"cm"))

ggsave(p10.1/p10.2,filename = "Figure2_B.png",width = 4,height =4,dpi = 1000)

###########C-D

p6 <- DimPlot(Trm,label = F,group.by = "celltype",cols = c("#8B4513","#FFA500"))+ggtitle("Trm")+

theme(aspect.ratio = 1,plot.title = element_text(hjust = 0.5),legend.position = "right",

axis.text = element_blank(),axis.ticks = element_blank(),text = element_text(size = 10),legend.key.height=unit(0.1,'cm'),legend.key.width =unit(0.2,'cm'))+

guides(colour = guide_legend(override.aes = list(size=1.8),ncol = 1))

p6.1 <- FeaturePlot(Trm,features = "CD4",cols = c("grey","red"),max.cutoff = 4.5)+NoLegend()+

theme(aspect.ratio = 1,plot.title = element_text(hjust = 0.5),axis.title = element_blank(),axis.line = element_blank(),

axis.text = element_blank(),axis.ticks = element_blank(),text = element_text(size = 10))

p6.2 <- FeaturePlot(Trm,features = "CD8A",cols = c("grey","red"),max.cutoff = 4.5)+

theme(aspect.ratio = 1,plot.title = element_text(hjust = 0.5),axis.title = element_blank(),axis.line = element_blank(),

axis.text = element_blank(),axis.ticks = element_blank(),text = element_text(size = 10))

p6+p6.1+p6.2

ggsave(p6+p6.1+p6.2,filename = "Figure2_C-D.png",width = 10,height = 3,dpi = 1000)

###########E

Trm$CellType <- str_c(Trm$Group,Trm$celltype,sep = " ")

table(Trm$CellType)

Trm@active.ident <- factor(Trm$CellType)

cluster.averages <- AverageExpression(Trm, return.seurat = T)

Tissue_residuce.gene <- c("ITGAE","CD69","ITGB2","ITGA4","ITGAL","ITGB7","ITGA1")

Migratie.gene <- c("CCR7","CCR4","CCR2","CCR10","CCR8","CXCR1","CXCR2","CXCR3","CXCR4","CXCR5",

"CCR6","CCR9","CCR1","CCR3","CX3CR1","CXCR6","CCR5","ACKR3","SELL","SELP","S1PR1","S1PR4","S1PR5")

s <- GetAssayData(cluster.averages, slot="scale.data")[c(Tissue_residuce.gene,Migratie.gene), ]

annotation_row = data.frame(

GeneClass = factor(rep(c("Intergrins", "Chemokine receptors","Selectins", "S1P receptors"), c(7, 18, 2,3)),levels = c("Intergrins", "Chemokine receptors","Selectins", "S1P receptors"))

)

rownames(annotation_row) = c(Tissue_residuce.gene,Migratie.gene)

ann_colors = list(

#GeneClass = c("Intergrins"="#9370DB","Chemokine receptors"="#1E90FF","Selectins"="#5F9EA0","S1P receptors"="#98FB98")

GeneClass = c("Intergrins"="chocolate","Chemokine receptors"="olivedrab","Selectins"="darkred","S1P receptors"="darkmagenta")

)

png(filename = "Figure2_E.png",width = 2.5*1000,height = 3.5*1000,res = 6*100)

p <- pheatmap(s, color = colorRampPalette(c("navy", "white", "firebrick3"))(21), border_color = "black", angle_col = 45, breaks=seq(-2.0,2.0,length.out=20), treeheight_row = 0,

cluster_rows = F,cluster_cols = F,gaps_row = c(7,25,27),gaps_col = 2,

annotation_row = annotation_row,annotation_names_row = F,annotation_colors = ann_colors,

fontsize_col = 10,fontsize_row = 10,fontsize = 9)

dev.off()

###########F

compaired <- list(c("CD4 Trm","CD8 Trm"))

data <- FetchData(Trm,vars = c("CellType","Proliferation_score1"))

data <- subset(data,CellType=="B-ALL CD4 Trm"|CellType=="B-ALL CD8 Trm")

data$CellType <- str_replace_all(data$CellType,"B-ALL CD4 Trm","CD4 Trm")

data$CellType <- str_replace_all(data$CellType,"B-ALL CD8 Trm","CD8 Trm")

p8.1 <- ggplot(data,aes(x=CellType,y=Proliferation_score1,fill=CellType))+geom_violin()+theme_classic()+ggtitle("Proliferation score")+scale_x_discrete("")+scale_fill_manual(values = c("#8B4513","#FFA500"))+

theme(title = element_text(face = "plain",size = 12),

legend.title = element_blank(),

legend.text = element_text(size = 12),

legend.key.size = unit(0.5, 'cm'), #change legend key size

aspect.ratio = 1.2,

axis.text.x = element_blank(),

axis.text.y = element_text(size = 10),

axis.title.x = element_blank(),

axis.title.y = element_blank(),

panel.border = element_blank(),

axis.ticks.x = element_blank(),

plot.title=element_text(hjust = 0.5,vjust = 5) )+

geom_boxplot(width=0.2,position=position_dodge(0.9),outlier.colour = NA,fill="white")+

geom_signif(comparisons = compaired,

step_increase = 0.2,

map_signif_level = T,

test = wilcox.test,textsize = 3.5)+NoLegend()

data <- FetchData(Trm,vars = c("CellType","Cytotoxicity_score1"))

data <- subset(data,CellType=="B-ALL CD4 Trm"|CellType=="B-ALL CD8 Trm")

data$CellType <- str_replace_all(data$CellType,"B-ALL CD4 Trm","CD4 Trm")

data$CellType <- str_replace_all(data$CellType,"B-ALL CD8 Trm","CD8 Trm")

p8.2 <- ggplot(data,aes(x=CellType,y=Cytotoxicity_score1,fill=CellType))+geom_violin()+theme_classic()+ggtitle("Cytotoxicity score")+scale_x_discrete("")+scale_fill_manual(values = c("#8B4513","#FFA500"))+

theme(title = element_text(face = "plain",size = 12),

legend.title = element_blank(),

legend.text = element_text(size = 12),

legend.key.size = unit(0.5, 'cm'), #change legend key size

aspect.ratio = 1.2,

axis.text.x = element_blank(),

axis.text.y = element_text(size = 10),

axis.title.x = element_blank(),

axis.title.y = element_blank(),

panel.border = element_blank(),

axis.ticks.x = element_blank(),

plot.title=element_text(hjust = 0.5,vjust = 5) )+

geom_boxplot(width=0.2,position=position_dodge(0.9),outlier.colour = NA,fill="white")+

geom_signif(comparisons = compaired,

step_increase = 0.2,

map_signif_level = T,

test = wilcox.test,textsize = 3.5)+NoLegend()

data <- FetchData(Trm,vars = c("CellType","Exhausted_score1"))

data <- subset(data,CellType=="B-ALL CD4 Trm"|CellType=="B-ALL CD8 Trm")

data$CellType <- str_replace_all(data$CellType,"B-ALL CD4 Trm","CD4 Trm")

data$CellType <- str_replace_all(data$CellType,"B-ALL CD8 Trm","CD8 Trm")

p8.3 <- ggplot(data,aes(x=CellType,y=Exhausted_score1,fill=CellType))+geom_violin()+theme_classic()+ggtitle("Exhausted score")+scale_x_discrete("")+scale_fill_manual(values = c("#8B4513","#FFA500"))+

theme(title = element_text(face = "plain",size = 12),

legend.title = element_blank(),

legend.text = element_text(size = 12),

legend.key.size = unit(0.5, 'cm'), #change legend key size

aspect.ratio = 1.2,

axis.text.x = element_blank(),

axis.text.y = element_text(size = 10),

axis.title.x = element_blank(),

axis.title.y = element_blank(),

panel.border = element_blank(),

axis.ticks.x = element_blank(),

plot.title=element_text(hjust = 0.5,vjust = 5) )+

geom_boxplot(width=0.2,position=position_dodge(0.9),outlier.colour = NA,fill="white")+

geom_signif(comparisons = compaired,

step_increase = 0.2,

map_signif_level = T,

test = wilcox.test,textsize = 3.5)+NoLegend()

data <- FetchData(Trm,vars = c("CellType","Tissue_residuce_score1"))

data <- subset(data,CellType=="B-ALL CD4 Trm"|CellType=="B-ALL CD8 Trm")

data$CellType <- str_replace_all(data$CellType,"B-ALL CD4 Trm","CD4 Trm")

data$CellType <- str_replace_all(data$CellType,"B-ALL CD8 Trm","CD8 Trm")

p8.4 <- ggplot(data,aes(x=CellType,y=Tissue_residuce_score1,fill=CellType))+geom_violin()+theme_classic()+ggtitle("Tissue-resident score")+scale_x_discrete("")+scale_fill_manual(values = c("#8B4513","#FFA500"))+

theme(title = element_text(face = "plain",size = 12),

legend.title = element_blank(),

legend.text = element_text(size = 12),

legend.key.size = unit(0.5, 'cm'), #change legend key size

aspect.ratio = 1.2,

axis.text.x = element_blank(),

axis.text.y = element_text(size = 10),

axis.title.x = element_blank(),

axis.title.y = element_blank(),

panel.border = element_blank(),

axis.ticks.x = element_blank(),

plot.title=element_text(hjust = 0.5,vjust = 5) )+

geom_boxplot(width=0.2,position=position_dodge(0.9),outlier.colour = NA,fill="white")+

geom_signif(comparisons = compaired,

step_increase = 0.2,

map_signif_level = T,

test = wilcox.test,textsize = 3.5)

#ggsave(plot = p8.1+p8.2+p8.3+p8.4+plot_layout(nrow = 1),filename = "8.png",width = 13,height = 5,dpi = 1000)

ggsave(plot = p8.1+p8.2+p8.3+p8.4+plot_layout(nrow = 1),filename = "Figure2_F.png",width = 13,height = 5,dpi = 1000)

############Figure3#################

###########A

scRNA1$clone.status[is.na(scRNA1$clone.status)] <- "Non-TCR detected"

data <- data.frame(UMAP_1=c(-1,5.5,6.5,4),UMAP_2=c(-3.2,-7,5.5,2),celltype=c("Tem","Tff","Trm","Tex"))

p15 <- DimPlot(scRNA1,group.by = "clone.status",cols = c("red","blue","grey"))+

geom_text(aes(x =UMAP_1,y = UMAP_2, label=celltype),data,size=3)+

guides(colour = guide_legend(override.aes = list(size=3),ncol = 1))+ggtitle("clone status of B-ALL")+

theme(aspect.ratio = 1,plot.title = element_text(hjust = 0.5),legend.position = "right",legend.key.height=unit(0.1,'cm'),legend.key.width =unit(0.2,'cm'),

axis.text = element_blank(),axis.ticks = element_blank(),text = element_text(size = 10))+

guides(colour = guide_legend(override.aes = list(size=1.8),ncol = 1))

ggsave(plot = p15,filename = "Figure3_A.png",width = 5,height = 3,dpi = 1000)

###########B

ALL_Trm <- subset(Trm,Group=="B-ALL")

scRNA1$CellType <- factor(scRNA1$CellType,levels = c("CD4 Naive","CD4 Tff","CD4 Trm","CD4 Tex","CD8 Naive","CD8 Tem","CD8 Tff","CD8 Trm","CD8 Tex","Tcm","Th","Treg","MAIT","γδ T"))

scRNA1$CellType[colnames(subset(ALL_Trm,celltype=="CD4 Trm"))] <- "CD4 Trm"

scRNA1$CellType[colnames(subset(ALL_Trm,celltype=="CD8 Trm"))] <- "CD8 Trm"

in.data <- FetchData(scRNA1,vars = c("Cell_Name","clone.id","clone.status","sample","CellType"))

in.data <- na.omit(in.data)

colnames(in.data)[c(4,5)] <- c("patient","majorCluster")

in.data$loc <- "PB"

in.data <- subset(in.data,majorCluster!=c("γδ T"))

in.data$majorCluster <- factor(in.data$majorCluster,

levels =c("CD4 Naive","CD4 Tff","CD4 Trm","CD4 Tex","CD8 Naive","CD8 Tem","CD8 Tff","CD8 Trm","CD8 Tex","Tcm","Th","Treg","MAIT"))

png("Figure3_B.png",width = 4*1000,height =4*1000,res = 6*100)

{

y1 <- c()

for (i in levels(in.data$majorCluster)){

k1 <- subset(in.data,majorCluster== i )

x <- c()

for (j in levels(in.data$majorCluster)){

k2 <- subset(in.data,majorCluster==j)

x1 <- length(na.omit(match(k1$clone.id,k2$clone.id))) / length(k1$clone.id)

x <- c(x,x1)

}

y1 <- rbind(y1,x)

}

colnames(y1) <- levels(in.data$majorCluster)

rownames(y1) <- levels(in.data$majorCluster)

}

y1 <- round(y1,2)

y1 <- y1[,rev(c(1:13))]

p <- pheatmap::pheatmap(y1,cluster_rows = F,cluster_cols = F,

#color = colorRampPalette(c("royalblue","lavender","white"))(20),

color = colorRampPalette(c("#FFECEC","#FF5151","#AE0000"))(21),

fontsize = 14,fontsize_row = 16,fontsize_col = 16,angle_col = 90,number_format = "%.2g",

display_numbers = TRUE,number_color = "black",legend = T,legend_breaks =seq(0,1,0.5),legend_labels = c("0","0.5","1"))

dev.off()

###########C-F

in.data1 <- subset(in.data,majorCluster=="CD4 Naive"|majorCluster=="CD4 Tff"|majorCluster=='CD4 Trm'|majorCluster=="CD4 Tex")

in.data1$majorCluster <- factor(in.data1$majorCluster,levels = c("CD4 Naive","CD4 Tff","CD4 Trm","CD4 Tex"))

in.data2 <- subset(in.data,majorCluster=="CD8 Naive"|majorCluster=="CD8 Tem"|majorCluster=="CD8 Tff"|majorCluster=="CD8 Trm"|majorCluster=="CD8 Tex")

in.data2$majorCluster <- factor(in.data2$majorCluster,levels = c("CD8 Naive","CD8 Tem","CD8 Tff","CD8 Trm","CD8 Tex"))

out.data1 <- Startrac.run(in.data1, proj="B-ALL", cores=2,verbose=F)

out.data2 <- Startrac.run(in.data2, proj="B-ALL", cores=2,verbose=F)

x1 <- out.data1@cluster.data[-c(1:4),]

colnames(x1) <- c('patient',"CellType","Expansion","Migration","Transition")

x1$CellType <- factor(x1$CellType,levels = c("CD4 Naive","CD4 Tff","CD4 Trm","CD4 Tex"))

p17 <- ggplot(x1,aes(x=CellType, y=Expansion)) +geom_boxplot(colour = "darkred") +xlab("")+geom_point(colour = "darkred")+theme_classic()+

theme(text=element_text(size=18),

title = element_text(face = "bold"),

legend.title = element_text(size = 18,face = "plain"),

aspect.ratio = 0.8,

axis.text.x = element_text(angle = 45, vjust =1.0, hjust=1.0,colour = "black"),

axis.text.y = element_text(hjust = 0.5, vjust = 0.5,colour = "black"),

plot.title=element_text(hjust = 0.5))

p18 <- ggplot(x1,aes(x=CellType, y=Transition)) +geom_boxplot(colour = "darkgreen") +xlab("")+geom_point(colour = "darkgreen")+theme_classic()+

theme(text=element_text(size=18),

title = element_text(face = "bold"),

legend.title = element_text(size = 18,face = "plain"),

aspect.ratio = 0.8,

axis.text.x = element_text(angle = 45, vjust =1.0, hjust=1.0,colour = "black"),

axis.text.y = element_text(hjust = 0.5, vjust = 0.5,colour = "black"),

plot.title=element_text(hjust = 0.5))

png("Figure3_C.png",width = 50*100,height = 25*100,res = 6*100)

p17+p18

dev.off()

png("Figure3_E.png",width = 25*100,height = 20*100,res = 6*100)

Startrac::plot(out.data1,index.type="pairwise.tran",byPatient=T)

dev.off()

x2 <- out.data2@cluster.data[-c(1:5),]

colnames(x2) <- c('patient',"CellType","Expansion","Migration","Transition")

x2$CellType <- factor(x2$CellType,levels = c("CD8 Naive","CD8 Tem","CD8 Tff","CD8 Trm","CD8 Tex"))

p19 <- ggplot(x2,aes(x=CellType, y=Expansion)) +geom_boxplot(colour = "darkred") +xlab("")+geom_point(colour = "darkred")+theme_classic()+

theme(text=element_text(size=18),

title = element_text(face = "bold"),

legend.title = element_text(size = 18,face = "plain"),

aspect.ratio = 0.8,

axis.text.x = element_text(angle = 45, vjust =1.0, hjust=1.0,colour = "black"),

axis.text.y = element_text(hjust = 0.5, vjust = 0.5,colour = "black"),

plot.title=element_text(hjust = 0.5))

p20 <- ggplot(x2,aes(x=CellType, y=Transition)) +geom_boxplot(colour = "darkgreen") +xlab("")+geom_point(colour = "darkgreen")+theme_classic()+

theme(text=element_text(size=18,colour = "black"),

title = element_text(face = "bold"),

legend.title = element_text(size = 18,face = "plain"),

aspect.ratio = 0.8,

axis.text.x = element_text(angle = 45, vjust =1.0, hjust=1.0,colour = "black"),

axis.text.y = element_text(hjust = 0.5, vjust = 0.5,colour = "black"),

plot.title=element_text(hjust = 0.5))

png("Figure3_D.png",width = 50*100,height = 25*100,res = 6*100)

p19+p20

dev.off()

png("Figure3_F.png",width = 25*100,height = 20*100,res = 6*100)

Startrac::plot(out.data2,index.type="pairwise.tran",byPatient=T)

dev.off()

############Figure4#################

load("Monocle2.Rdata")

###########A

p1 = plot_cell_trajectory(mycds2, color_by = "State")+theme_classic()+

theme(legend.position = "right",aspect.ratio = 0.7,text = element_text(size = 16),

legend.text = element_text(size = 16),legend.title =element_text(size = 16) ,

axis.title =element_blank() ,axis.text = element_blank(),axis.line =element_blank(),axis.ticks = element_blank())+

guides(color=guide_legend(override.aes = list(size=5)))

data<- as.data.frame(t(mycds2@reducedDimS))

colnames(data) <- c("Component_1","Component_2")

data$celltype <- mycds2$CellType

cell_type_med <- data %>%

group_by(celltype) %>%

summarise(

"Component_1" = median(Component_1),

"Component_2" = median(Component_2)

)

cell_type_med$Component_1[1] <- 6.5

cell_type_med$Component_1[2] <- -11

cell_type_med$Component_2[2] <- -3.5

cell_type_med$Component_1[3] <- -3

cell_type_med$Component_2[3] <- 5

p3 <- plot_cell_trajectory(mycds2, color_by = "CellType")+theme_classic()+

scale_color_manual(values= c("#1E90FF","#8B4513","#D2B48C")) +

geom_text(aes(x =Component_1,y = Component_2, label=celltype),cell_type_med,size=6)+

theme(legend.position = "right",aspect.ratio = 0.7,text = element_text(size = 16),

legend.text = element_text(size = 16),legend.key.size =unit(0.5,"cm") ,legend.title =element_text(size = 16) ,

axis.title =element_blank() ,axis.text = element_blank(),axis.line =element_blank(),axis.ticks = element_blank())+

guides(color=guide_legend(override.aes = list(size=5)))

p6 <- plot_cell_trajectory(mycds2, color_by = "Pseudotime")+theme_classic()+scale_color_viridis(option = "B")+

theme(legend.position = "right",legend.key.height = unit(0.7,"cm"),legend.key.width = unit(0.8,"cm"),

aspect.ratio = 0.7,text = element_text(size = 16),

legend.text = element_text(size = 16),legend.title =element_text(size = 16),

axis.title =element_blank() ,axis.text = element_blank(),axis.line =element_blank(),axis.ticks = element_blank())

p3/p1/p6

ggsave(p3/p1/p6,filename = "Figure4_A.png",width = 10,height = 14,dpi = 1000)

###########B

p1 = plot_cell_trajectory(mycds, color_by = "State")+theme_classic()+

theme(legend.position = "right",aspect.ratio = 0.7,text = element_text(size = 16),

legend.text = element_text(size = 16),legend.title =element_text(size = 16) ,

axis.title =element_blank() ,axis.text = element_blank(),axis.line =element_blank(),axis.ticks = element_blank())+

guides(color=guide_legend(override.aes = list(size=5)))

data<- as.data.frame(t(mycds@reducedDimS))

colnames(data) <- c("Component_1","Component_2")

data$celltype <- mycds$CellType

cell_type_med <- data %>%

group_by(celltype) %>%

summarise(

"Component_1" = median(Component_1),

"Component_2" = median(Component_2)

)

cell_type_med$Component_1[1] <- 5

cell_type_med$Component_1[2] <- 8.5

cell_type_med$Component_2[2] <- 0

cell_type_med$Component_1[3] <- -11

cell_type_med$Component_1[4] <- -4

cell_type_med$Component_2[4] <- 5

p3 <- plot_cell_trajectory(mycds, color_by = "CellType")+theme_classic()+

scale_color_manual(values=c("#FFFF00","#808000","#FFA500","#D2691E")) +

geom_text(aes(x =Component_1,y = Component_2, label=celltype),cell_type_med,size=6)+

theme(legend.position = "right",aspect.ratio = 0.7,text = element_text(size = 16),

legend.text = element_text(size = 16),legend.key.size =unit(0.5,"cm") ,legend.title =element_text(size = 16) ,

axis.title =element_blank() ,axis.text = element_blank(),axis.line =element_blank(),axis.ticks = element_blank())+

guides(color=guide_legend(override.aes = list(size=5)))

p6 <- plot_cell_trajectory(mycds, color_by = "Pseudotime")+theme_classic()+scale_color_viridis(option = "B")+

theme(legend.position = "right",

legend.key.height = unit(0.7,"cm"),legend.key.width = unit(0.8,"cm"),

aspect.ratio = 0.7,text = element_text(size = 16),

legend.text = element_text(size = 16),legend.title =element_text(size = 16),

axis.title =element_blank() ,axis.text = element_blank(),axis.line =element_blank(),axis.ticks = element_blank())

p3/p1/p6

ggsave(p3/p1/p6,filename = "Figure4_B.png",width = 10,height = 14,dpi = 1000)

###########C

CD4_marker <- CD4state.markers

CD4_marker$State <- "CD4"

CD4_marker$cluster <- str_c("CD4",CD4_marker$cluster,sep = "_")

CD8_marker <-CD8state.markers

CD8_marker$State <- "CD8"

CD8_marker$cluster <- str_c("CD8",CD8_marker$cluster,sep = "_")

markers <- rbind(CD4_marker,CD8_marker)

markers$p_val_adj1 <- "P-val adj < 0.01"

markers$p_val_adj1[match(rownames(markers %>% filter(p_val_adj >= 0.01)),rownames(markers))] <- "P-val adj ≥ 0.01"

maxmarkers <- markers %>% group_by(cluster) %>% slice_max(n = 5, order_by = avg_log2FC)

maxmarkers$gene <- str_c("italic(",maxmarkers$gene,sep = "'")

maxmarkers$gene <- str_c(maxmarkers$gene,"')",sep = "")

minmarkers <- markers %>% group_by(cluster) %>% slice_min(n = 5, order_by = avg_log2FC)

minmarkers$gene <- str_c("italic(",minmarkers$gene,sep = "'")

minmarkers$gene <- str_c(minmarkers$gene,"')",sep = "")

dat<-data.frame(x=c("CD4_1","CD4_2","CD4_3","CD8_1","CD8_2","CD8_3"),

y=0,

label=c(1,2,3,1,2,3),State=c("CD4","CD4","CD4","CD8","CD8","CD8"))

datbar<-data.frame(x=c("CD4_1","CD4_2","CD4_3","CD8_1","CD8_2","CD8_3"),

y=c(6,4.5,3,6,5,3))

p23 <- ggplot()+

geom_col(data=datbar,aes(x=x,y=c(6,2.7,4.7,5.7,3,5)),fill="grey",alpha=0.2)+

geom_col(data=datbar,aes(x=x,y=-c(4,4.5,5.5,5,5,4.3)),fill="grey",alpha=0.2)+

geom_jitter(data=markers %>% filter(p_val_adj1 == "P-val adj < 0.01"),

aes(x=cluster,y=avg_log2FC,

color=p_val_adj1),size=0.5,show.legend = T)+

geom_jitter(data=markers %>% filter(p_val_adj1 == "P-val adj ≥ 0.01"),

aes(x=cluster,y=avg_log2FC,

color=p_val_adj1),size=0.5,show.legend = T)+

scale_color_manual(name=NULL,

values = c("red","darkgrey"))+

ggnewscale::new_scale_fill()+

geom_text_repel(data = maxmarkers,mapping = aes(x=cluster,y=avg_log2FC, label = gene),size=2.5,parse = TRUE)+

geom_text_repel(data = minmarkers,mapping = aes(x=cluster,y=avg_log2FC, label = gene),size=2.5,parse = TRUE)+

theme_minimal()+scale_y_continuous(breaks = c(6,3,1,0,-1,-3,-5))+

theme(

axis.line.y = element_line(),

axis.ticks.y = element_line(),

panel.grid = element_blank(),

legend.position = "right",

legend.text = element_text(size = 12),

#legend.box = "vertical",

#legend.box.just = "top",

#legend.justification = c(1,1),

legend.direction = "vertical",

axis.text.x = element_blank(),

axis.title = element_text(size = 14))+

labs(x="State",y="Average logFC")+

geom_tile(data=dat,

aes(x=x,y=y,fill=State),

height=0.4,color="black",

alpha=0.9,

show.legend = T)+

scale_fill_manual(values = c("#4177aa","#a94698"))+

geom_text(data=dat,aes(x=x,y=y,label=label))+

geom_hline(yintercept=c(-1,1), linetype="longdash",alpha=0.7)

ggsave(p23,filename = "Figure4_C.png",width = 7,height = 6,dpi = 1000)

###########D

BEAM_res=BEAM_res[,c("gene_short_name","pval","qval")]

BEAM_genes <- top_n(BEAM_res, n = 100, dplyr::desc(qval)) %>% pull(gene_short_name) %>% as.character()

tmp1=plot_genes_branched_heatmap(mycds[BEAM_genes,],

branch_point = 1,

num_clusters = 3,

cores = 8,

branch_labels = c("State 2", "State 3"),

#hmcols = NULL,

#hmcols = colorRampPalette(rev(brewer.pal(9, "PRGn")))(62),

#hmcols = colorRampPalette(c("navy","white", "firebrick3"))(62),

branch_colors = c("#F8766D", "#00BA38", "#619CFF"),

use_gene_short_name = T,

show_rownames = T,

scale_max = 3,

scale_min = -3,

cluster_rows = T,

return_heatmap = T

)

png("Figure4_D.png",width = 30*100,height = 35*100,res = 6*100)

tmp1$ph_res

dev.off()

gene_State=tmp1$annotation_row

gene_State$gene=rownames(gene_State)

library(clusterProfiler)

library(org.Hs.eg.db)

allcluster_go=data.frame()

for (i in unique(gene_State$Cluster)) {

small_gene_State=filter(gene_State,gene_State$Cluster==i)

df_name=bitr(small_gene_State$gene, fromType="SYMBOL", toType=c("ENTREZID"), OrgDb="org.Hs.eg.db")

go <- enrichGO(gene = unique(df_name$ENTREZID),

OrgDb = org.Hs.eg.db,

keyType = 'ENTREZID',

ont = "BP",

pAdjustMethod = "BH",

pvalueCutoff = 0.05,

qvalueCutoff = 0.2,

readable = TRUE)

go_res=go@result

if (dim(go_res)[1] != 0) {

go_res$cluster=i

allcluster_go=rbind(allcluster_go,go_res)

}

}

head(allcluster_go[,c("ID","Description","qvalue","cluster")])

############Figure5#################

load("Survival.Rdata")

###########B

theme_kcl <- theme_bw() +

theme(panel.grid=element_blank(),

axis.text = element_text(color = "black"),

aspect.ratio = 1)

#####CD8_EFS

fit1 <- survfit(Surv(EFS,EFS_event) ~State1, data = CD8_survival)

survival1<- ggsurvplot(fit1,

data = CD8_survival,

risk.table =F,

pval = F,

conf.int= F,

risk.table.height = 0.5,

legend.title = "",

legend.lab=c("Low; n=68","High; n=69"),

legend = c(0.7,0.75),

#legend.labs = levels(lung[[i]]),##

surv.median.line = "hv",

palette="lancet",

pval.coord = c(2500,0.9)

)

plot1 <- survival1$plot+ylab("EFS")+xlab("Days")+ggtitle("State1")+theme_kcl+theme(axis.title.x = element_blank(),plot.title = element_text(size = 14,hjust = 0.5,vjust = 0),legend.text = element_text(size = 14))+NoLegend()+

annotate("text", x = 3000, y = 0.9, label = "p = 0.26",fontface=3,size=5)

fit2 <- survfit(Surv(EFS,EFS_event) ~State2, data = CD8_survival)

survival2<- ggsurvplot(fit2,

data = CD8_survival,

risk.table =F,

pval = F,

conf.int= F,

risk.table.height = 0.5,

legend.title = "",

legend.lab=c("high","low"),

legend = c(0.9,0.75),

#legend.labs = levels(lung[[i]]),##

surv.median.line = "hv",

palette="lancet",

pval.coord = c(2500,0.9))

plot2 <- survival2$plot+ylab("EFS")+xlab("Days")+ggtitle("State2")+theme_kcl+

theme(axis.text.y = element_blank(),axis.title.y = element_blank(),axis.ticks.y = element_blank(),plot.title = element_text(size = 14,hjust = 0.5,vjust = 0))+NoLegend()+

annotate("text", x = 3000, y = 0.9, label = "p = 0.028",fontface=3,size=5)

fit3 <- survfit(Surv(EFS,EFS_event) ~State3, data = CD8_survival)

survival3<- ggsurvplot(fit3,

data = CD8_survival,

risk.table =F,

pval = F,

conf.int= F,

risk.table.height = 0.5,

legend.title = "",

legend.lab=c("high","low"),

legend = c(0.9,0.75),

#legend.labs = levels(lung[[i]]),##

surv.median.line = "hv",

palette="lancet",

pval.coord = c(2500,0.9))

plot3 <- survival3$plot+ylab("EFS")+xlab("Days")+ggtitle("State3")+theme_kcl+

theme(axis.text.y = element_blank(),axis.title.y = element_blank(),axis.ticks.y = element_blank(),axis.title.x = element_blank(),plot.title = element_text(size = 14,hjust = 0.5,vjust = 0))+

NoLegend()+

annotate("text", x = 3000, y = 0.9, label = "p = 0.016",fontface=3,size=5)

###CD8_OS

fit4 <- survfit(Surv(OS,OS_event) ~State1, data = CD8_survival)

survival4<- ggsurvplot(fit4,

data = CD8_survival,

risk.table =F,

pval = F,

conf.int= F,

risk.table.height = 0.5,

legend.title = "",

legend.lab=c("high","low"),

legend = c(0.9,0.75),

#legend.labs = levels(lung[[i]]),##

surv.median.line = "hv",

palette="lancet",

pval.coord = c(2500,0.9))

plot4 <- survival4$plot+ylab("OS")+xlab("Days")+theme_kcl+theme(axis.title.x = element_blank())+NoLegend()+

annotate("text", x = 3000, y = 0.9, label = "p = 0.22",fontface=3,size=5)

fit5 <- survfit(Surv(OS,OS_event) ~State2, data = CD8_survival)

survival5<- ggsurvplot(fit5,

data = CD8_survival,

risk.table =F,

pval = F,

conf.int= F,

risk.table.height = 0.5,

legend.title = "",

legend.lab=c("high","low"),

legend = c(0.9,0.75),

#legend.labs = levels(lung[[i]]),##

surv.median.line = "hv",

palette="lancet",

pval.coord = c(2500,0.9))

plot5 <- survival5$plot+ylab("OS")+xlab("Days")+theme_kcl+

theme(axis.text.y = element_blank(),axis.title.y = element_blank(),axis.ticks.y = element_blank())+NoLegend()+

annotate("text", x = 3000, y = 0.9, label = "p = 0.017",fontface=3,size=5)

fit6 <- survfit(Surv(OS,OS_event) ~State3, data = CD8_survival)

survival6<- ggsurvplot(fit6,

data = CD8_survival,

risk.table =F,

pval = F,

conf.int= F,

risk.table.height = 0.5,

legend.title = "",

legend.lab=c("low","high"),

legend = c(0.9,0.75),

#legend.labs = levels(lung[[i]]),##

surv.median.line = "hv",

palette="lancet",

pval.coord = c(2500,0.9))

plot6 <- survival6$plot+ylab("OS")+xlab("Days")+theme_kcl+

theme(axis.text.y = element_blank(),axis.title.y = element_blank(),axis.ticks.y = element_blank(),axis.title.x = element_blank())+

NoLegend()+annotate("text", x = 3000, y = 0.9, label = "p = 0.14",fontface=3,size=5)

(plot1+plot2+plot3)/(plot4+plot5+plot6)

ggsave((plot1+plot2+plot3)/(plot4+plot5+plot6),filename = "Figure5_B.png",width = 9,height = 6.5,dpi = 1000)

############FigureS1#################

load("OH_Young_PBMC.Rdata")

###########A-B

markers<-c("CD3E","CD3D","CD3G","IL7R","CD8A","MS4A1","CD19","CD79A","GNLY", "NKG7", 'FCGR3A',"S100A8","S100A9",'LYZ','CD14','CST3','FCER1A','PPBP',"PF4","HBA1","HBA2")

p1 <- DimPlot(Health,group.by = "CellType",label = T,repel = T)+ggtitle("")+

DotPlot(Health,features=unique(markers),group.by = "celltype",

assay = "RNA")+

RotatedAxis()+

theme_bw()+

theme(text = element_text(size = 12),panel.background = element_blank(),panel.grid = element_blank(),

axis.text=element_text(size=12,color = "black"),axis.title=element_blank(),legend.position="right",legend.text=element_text(size=12),legend.title=element_text(size=12,face="bold"),

axis.text.x=element_text(angle = 90,hjust = 0,vjust = 1))+

scale_x_discrete(position = "top")

ggsave(p1,filename = "FigureS1_A-B.png",width = 15,height = 7,dpi = 1000)

###########C

Health@active.ident <- Health$CellType

Health.markers <- FindAllMarkers(Health, only.pos = TRUE, min.pct = 0.25, logfc.threshold = 0.25)

Health.topMarkers <- Health.markers %>% group_by(cluster) %>% slice_max(n = 6, order_by = avg_log2FC)

cluster.averages <- AverageExpression(Health, return.seurat = T)

s <- GetAssayData(cluster.averages, slot = "scale.data")[unique(Health.topMarkers$gene), ]

p2 <- DoHeatmap(cluster.averages,features = unique(Health.topMarkers$gene),raster = T,draw.lines = F,size = 3)+NoLegend()

ggsave(p2,filename = "FigureS1_C.png",width = 7,height =6.5,dpi = 1000)

############FigureS2#################

###########A-B

load("singleR.Rdata")

png("FigureS2_A.png",width = 40*100,height = 32*100,res = 6*100)

p <- pheatmap::pheatmap(mat = log2(aa1+10),border_color = F,color = viridis(21),legend = T,fontsize = 12)

dev.off()

png("FigureS2_B.png",width = 40*100,height = 32*100,res = 6*100)

p <- pheatmap::pheatmap(mat = log2(aa+10),border_color = F,color = viridis(21),legend = T,fontsize = 12)

dev.off()

###########C-D

#"CD3D","CD3G","CD4","CD8A","SELL","TCF7","CCR7","LEF1","NKG7","GNLY","PRF1","IFNG","TRDC","TRDV2","GZMK","GZMB","CD69","ITGAE","MKI67","PDCD1","LAG3","TIGIT","FOXP3","TRAV1-2"

p0 <- FeaturePlot(scRNA1,features = "PRF1",cols = c("grey","red"))+NoLegend()+

theme(aspect.ratio = 1,plot.title = element_text(hjust = 0.5),axis.title = element_blank(),axis.line = element_blank(),

axis.text = element_blank(),axis.ticks = element_blank(),text = element_text(size = 10))

p1 <- FeaturePlot(scRNA1,features = "CD3D",cols = c("grey","red"))+NoLegend()+

theme(aspect.ratio = 1,plot.title = element_text(hjust = 0.5),axis.title = element_blank(),axis.line = element_blank(),

axis.text = element_blank(),axis.ticks = element_blank(),text = element_text(size = 10))

p2 <- FeaturePlot(scRNA1,features = "CD4",cols = c("grey","red"))+NoLegend()+

theme(aspect.ratio = 1,plot.title = element_text(hjust = 0.5),axis.title = element_blank(),axis.line = element_blank(),

axis.text = element_blank(),axis.ticks = element_blank(),text = element_text(size = 10))

p3 <- FeaturePlot(scRNA1,features = "CD8A",cols = c("grey","red"))+NoLegend()+

theme(aspect.ratio = 1,plot.title = element_text(hjust = 0.5),axis.title = element_blank(),axis.line = element_blank(),

axis.text = element_blank(),axis.ticks = element_blank(),text = element_text(size = 10))

p4 <- FeaturePlot(scRNA1,features = "SELL",cols = c("grey","red"))+NoLegend()+

theme(aspect.ratio = 1,plot.title = element_text(hjust = 0.5),axis.title = element_blank(),axis.line = element_blank(),

axis.text = element_blank(),axis.ticks = element_blank(),text = element_text(size = 10))

p5 <- FeaturePlot(scRNA1,features = "TCF7",cols = c("grey","red"))+NoLegend()+

theme(aspect.ratio = 1,plot.title = element_text(hjust = 0.5),axis.title = element_blank(),axis.line = element_blank(),

axis.text = element_blank(),axis.ticks = element_blank(),text = element_text(size = 10))

p6 <- FeaturePlot(scRNA1,features = "CCR7",cols = c("grey","red"))+NoLegend()+

theme(aspect.ratio = 1,plot.title = element_text(hjust = 0.5),axis.title = element_blank(),axis.line = element_blank(),

axis.text = element_blank(),axis.ticks = element_blank(),text = element_text(size = 10))

p7 <- FeaturePlot(scRNA1,features = "LEF1",cols = c("grey","red"))+NoLegend()+

theme(aspect.ratio = 1,plot.title = element_text(hjust = 0.5),axis.title = element_blank(),axis.line = element_blank(),

axis.text = element_blank(),axis.ticks = element_blank(),text = element_text(size = 10))

p8 <- FeaturePlot(scRNA1,features = "NKG7",cols = c("grey","red"))+NoLegend()+

theme(aspect.ratio = 1,plot.title = element_text(hjust = 0.5),axis.title = element_blank(),axis.line = element_blank(),

axis.text = element_blank(),axis.ticks = element_blank(),text = element_text(size = 10))

p9 <- FeaturePlot(scRNA1,features = "GNLY",cols = c("grey","red"))+NoLegend()+

theme(aspect.ratio = 1,plot.title = element_text(hjust = 0.5),axis.title = element_blank(),axis.line = element_blank(),

axis.text = element_blank(),axis.ticks = element_blank(),text = element_text(size = 10))

p10 <- FeaturePlot(scRNA1,features = "TRDC",cols = c("grey","red"))+NoLegend()+

theme(aspect.ratio = 1,plot.title = element_text(hjust = 0.5),axis.title = element_blank(),axis.line = element_blank(),

axis.text = element_blank(),axis.ticks = element_blank(),text = element_text(size = 10))

p11 <- FeaturePlot(scRNA1,features = "TRDV2",cols = c("grey","red"))+NoLegend()+

theme(aspect.ratio = 1,plot.title = element_text(hjust = 0.5),axis.title = element_blank(),axis.line = element_blank(),

axis.text = element_blank(),axis.ticks = element_blank(),text = element_text(size = 10))

p12 <- FeaturePlot(scRNA1,features = "CD69",cols = c("grey","red"))+NoLegend()+

theme(aspect.ratio = 1,plot.title = element_text(hjust = 0.5),axis.title = element_blank(),axis.line = element_blank(),

axis.text = element_blank(),axis.ticks = element_blank(),text = element_text(size = 10))

p13 <- FeaturePlot(scRNA1,features = "ITGAE",cols = c("grey","red"))+NoLegend()+

theme(aspect.ratio = 1,plot.title = element_text(hjust = 0.5),axis.title = element_blank(),axis.line = element_blank(),

axis.text = element_blank(),axis.ticks = element_blank(),text = element_text(size = 10))

p14 <- FeaturePlot(scRNA1,features = "MKI67",cols = c("grey","red"))+NoLegend()+

theme(aspect.ratio = 1,plot.title = element_text(hjust = 0.5),axis.title = element_blank(),axis.line = element_blank(),

axis.text = element_blank(),axis.ticks = element_blank(),text = element_text(size = 10))

p15 <- FeaturePlot(scRNA1,features = "PDCD1",cols = c("grey","red"))+NoLegend()+

theme(aspect.ratio = 1,plot.title = element_text(hjust = 0.5),axis.title = element_blank(),axis.line = element_blank(),

axis.text = element_blank(),axis.ticks = element_blank(),text = element_text(size = 10))

p16 <- FeaturePlot(scRNA1,features = "LAG3",cols = c("grey","red"))+NoLegend()+

theme(aspect.ratio = 1,plot.title = element_text(hjust = 0.5),axis.title = element_blank(),axis.line = element_blank(),

axis.text = element_blank(),axis.ticks = element_blank(),text = element_text(size = 10))

p17 <- FeaturePlot(scRNA1,features = "TIGIT",cols = c("grey","red"))+NoLegend()+

theme(aspect.ratio = 1,plot.title = element_text(hjust = 0.5),axis.title = element_blank(),axis.line = element_blank(),

axis.text = element_blank(),axis.ticks = element_blank(),text = element_text(size = 10))

p18 <- FeaturePlot(scRNA1,features = "FOXP3",cols = c("grey","red"))+NoLegend()+

theme(aspect.ratio = 1,plot.title = element_text(hjust = 0.5),axis.title = element_blank(),axis.line = element_blank(),

axis.text = element_blank(),axis.ticks = element_blank(),text = element_text(size = 10))

p19 <- FeaturePlot(scRNA1,features ="TRAV1-2",cols = c("grey","red"))+NoLegend()+

theme(aspect.ratio = 1,plot.title = element_text(hjust = 0.5),axis.title = element_blank(),axis.line = element_blank(),

axis.text = element_blank(),axis.ticks = element_blank(),text = element_text(size = 10))

ggsave(p1+p2+p3+p4+p5+p6+p7+p8+p9+p0+p10+p11+p12+p13+p14+p15+p16+p17+p18+p19+plot_layout(ncol = 4,nrow = 5),filename = "FigureS2_C.png",

width = 8,height =10,dpi = 1000)

p0 <- FeaturePlot(merges,features = "PRF1",cols = c("grey","red"))+NoLegend()+

theme(aspect.ratio = 1,plot.title = element_text(hjust = 0.5),axis.title = element_blank(),axis.line = element_blank(),

axis.text = element_blank(),axis.ticks = element_blank(),text = element_text(size = 10))

p1 <- FeaturePlot(merges,features = "CD3D",cols = c("grey","red"))+NoLegend()+

theme(aspect.ratio = 1,plot.title = element_text(hjust = 0.5),axis.title = element_blank(),axis.line = element_blank(),

axis.text = element_blank(),axis.ticks = element_blank(),text = element_text(size = 10))

p2 <- FeaturePlot(merges,features = "CD4",cols = c("grey","red"))+NoLegend()+

theme(aspect.ratio = 1,plot.title = element_text(hjust = 0.5),axis.title = element_blank(),axis.line = element_blank(),

axis.text = element_blank(),axis.ticks = element_blank(),text = element_text(size = 10))

p3 <- FeaturePlot(merges,features = "CD8A",cols = c("grey","red"))+NoLegend()+

theme(aspect.ratio = 1,plot.title = element_text(hjust = 0.5),axis.title = element_blank(),axis.line = element_blank(),

axis.text = element_blank(),axis.ticks = element_blank(),text = element_text(size = 10))

p4 <- FeaturePlot(merges,features = "SELL",cols = c("grey","red"))+NoLegend()+

theme(aspect.ratio = 1,plot.title = element_text(hjust = 0.5),axis.title = element_blank(),axis.line = element_blank(),

axis.text = element_blank(),axis.ticks = element_blank(),text = element_text(size = 10))

p5 <- FeaturePlot(merges,features = "TCF7",cols = c("grey","red"))+NoLegend()+

theme(aspect.ratio = 1,plot.title = element_text(hjust = 0.5),axis.title = element_blank(),axis.line = element_blank(),

axis.text = element_blank(),axis.ticks = element_blank(),text = element_text(size = 10))

p6 <- FeaturePlot(merges,features = "CCR7",cols = c("grey","red"))+NoLegend()+

theme(aspect.ratio = 1,plot.title = element_text(hjust = 0.5),axis.title = element_blank(),axis.line = element_blank(),

axis.text = element_blank(),axis.ticks = element_blank(),text = element_text(size = 10))

p7 <- FeaturePlot(merges,features = "LEF1",cols = c("grey","red"))+NoLegend()+

theme(aspect.ratio = 1,plot.title = element_text(hjust = 0.5),axis.title = element_blank(),axis.line = element_blank(),

axis.text = element_blank(),axis.ticks = element_blank(),text = element_text(size = 10))

p8 <- FeaturePlot(merges,features = "NKG7",cols = c("grey","red"))+NoLegend()+

theme(aspect.ratio = 1,plot.title = element_text(hjust = 0.5),axis.title = element_blank(),axis.line = element_blank(),

axis.text = element_blank(),axis.ticks = element_blank(),text = element_text(size = 10))

p9 <- FeaturePlot(merges,features = "GNLY",cols = c("grey","red"))+NoLegend()+

theme(aspect.ratio = 1,plot.title = element_text(hjust = 0.5),axis.title = element_blank(),axis.line = element_blank(),

axis.text = element_blank(),axis.ticks = element_blank(),text = element_text(size = 10))

p10 <- FeaturePlot(merges,features = "TRDC",cols = c("grey","red"))+NoLegend()+

theme(aspect.ratio = 1,plot.title = element_text(hjust = 0.5),axis.title = element_blank(),axis.line = element_blank(),

axis.text = element_blank(),axis.ticks = element_blank(),text = element_text(size = 10))

p11 <- FeaturePlot(merges,features = "TRDV2",cols = c("grey","red"))+NoLegend()+

theme(aspect.ratio = 1,plot.title = element_text(hjust = 0.5),axis.title = element_blank(),axis.line = element_blank(),

axis.text = element_blank(),axis.ticks = element_blank(),text = element_text(size = 10))

p12 <- FeaturePlot(merges,features = "CD69",cols = c("grey","red"))+NoLegend()+

theme(aspect.ratio = 1,plot.title = element_text(hjust = 0.5),axis.title = element_blank(),axis.line = element_blank(),

axis.text = element_blank(),axis.ticks = element_blank(),text = element_text(size = 10))

p13 <- FeaturePlot(merges,features = "ITGAE",cols = c("grey","red"))+NoLegend()+

theme(aspect.ratio = 1,plot.title = element_text(hjust = 0.5),axis.title = element_blank(),axis.line = element_blank(),

axis.text = element_blank(),axis.ticks = element_blank(),text = element_text(size = 10))

p14 <- FeaturePlot(merges,features = "MKI67",cols = c("grey","red"))+NoLegend()+

theme(aspect.ratio = 1,plot.title = element_text(hjust = 0.5),axis.title = element_blank(),axis.line = element_blank(),

axis.text = element_blank(),axis.ticks = element_blank(),text = element_text(size = 10))

p15 <- FeaturePlot(merges,features = "PDCD1",cols = c("grey","red"))+NoLegend()+

theme(aspect.ratio = 1,plot.title = element_text(hjust = 0.5),axis.title = element_blank(),axis.line = element_blank(),

axis.text = element_blank(),axis.ticks = element_blank(),text = element_text(size = 10))

p16 <- FeaturePlot(merges,features = "LAG3",cols = c("grey","red"))+NoLegend()+

theme(aspect.ratio = 1,plot.title = element_text(hjust = 0.5),axis.title = element_blank(),axis.line = element_blank(),

axis.text = element_blank(),axis.ticks = element_blank(),text = element_text(size = 10))

p17 <- FeaturePlot(merges,features = "TIGIT",cols = c("grey","red"))+NoLegend()+

theme(aspect.ratio = 1,plot.title = element_text(hjust = 0.5),axis.title = element_blank(),axis.line = element_blank(),

axis.text = element_blank(),axis.ticks = element_blank(),text = element_text(size = 10))

p18 <- FeaturePlot(merges,features = "FOXP3",cols = c("grey","red"))+NoLegend()+

theme(aspect.ratio = 1,plot.title = element_text(hjust = 0.5),axis.title = element_blank(),axis.line = element_blank(),

axis.text = element_blank(),axis.ticks = element_blank(),text = element_text(size = 10))

p19 <- FeaturePlot(merges,features ="TRAV1-2",cols = c("grey","red"))+NoLegend()+

theme(aspect.ratio = 1,plot.title = element_text(hjust = 0.5),axis.title = element_blank(),axis.line = element_blank(),

axis.text = element_blank(),axis.ticks = element_blank(),text = element_text(size = 10))

ggsave(p1+p2+p3+p4+p5+p6+p7+p8+p9+p0+p10+p11+p12+p13+p14+p15+p16+p17+p18+p19+plot_layout(ncol = 4,nrow = 5),filename = "FigureS2_D.png",

width = 8,height =10,dpi = 1000)

############FigureS4#################

###########A

x1 <- as.data.frame(prop.table(table(scRNAsub2$State,scRNAsub2$CellType),margin = 1))

colnames(x1) <- c('State','CellType','Ratio')

x1 <- subset(x1,CellType=="CD4 Trm"|CellType=="CD4 Tex"|CellType=="CD4 Tff")

x1 <- subset(x1,State!=4)

x1$Ratio <- round(x1$Ratio,2)

x1 <- dcast(x1,CellType~State)

colnames(x1)[2:4] <- c("State1","State2","State3")

x1$CellType <- as.character(x1$CellType)

x1 <- rbind(x1,c("Total","1","1","1"))

x1 <- rbind(x1,c(" "," "," "," "))

colnames(x1)[1] <- " "

g <- tableGrob(x1,rows = NULL)

#grid.newpage()

png(filename = "FigureS4_A.png",width = 40*100,height = 20*100,res = 10*100)

grid.draw(g)

dev.off()

x1.1 <- as.data.frame(table(scRNAsub2$State,scRNAsub2$CellType))

colnames(x1.1) <- c('State','CellType','Ratio')

x1.1 <- subset(x1.1,CellType=="CD4 Trm"|CellType=="CD4 Tex"|CellType=="CD4 Tff")

x1.1 <- subset(x1.1,State!=4)

x1.1 <- dcast(x1.1,CellType~State)

rownames(x1.1) <- x1.1$CellType

x1.1 <- x1.1[,-1]

chisq.test(x1.1$`1`)

chisq.test(x1.1$`2`)

chisq.test(x1.1$`3`)

###########B

x2 <- as.data.frame(prop.table(table(scRNAsub$State,scRNAsub$CellType),margin = 1))

colnames(x2) <- c('State','CellType','Ratio')

x2 <- subset(x2,CellType=="CD8 Trm"|CellType=="CD8 Tex"|CellType=="CD8 Tff"|CellType=="CD8 Tem")

x2$Ratio <- round(x2$Ratio,2)

x2 <- dcast(x2,CellType~State)

colnames(x2)[2:4] <- c("State1","State2","State3")

x2$CellType <- as.character(x2$CellType)

x2$State1[2] <- "0.002"

x2$State1[3] <- "0.744"

x2$State1[4] <- "0.254"

x2 <- rbind(x2,c("Total","1","1","1"))

x2 <- rbind(x2,c(" "," "," "," "))

colnames(x2)[1] <- " "

g2 <- tableGrob(x2,rows = NULL)

png(filename = "FigureS4_B.png",width = 40*100,height = 20*100,res = 10*100)

grid.draw(g2)

dev.off()

x2.1 <- as.data.frame(table(scRNAsub$State,scRNAsub$CellType))

colnames(x2.1) <- c('State','CellType','Ratio')

x2.1 <- subset(x2.1,CellType=="CD8 Trm"|CellType=="CD8 Tex"|CellType=="CD8 Tff"|CellType=="CD8 Tem")

x2.1 <- dcast(x2.1,CellType~State)

rownames(x2.1) <- x2.1$CellType

x2.1 <- x2.1[,-1]

chisq.test(x2.1$`1`)

chisq.test(x2.1$`2`)

chisq.test(x2.1$`1`)

###########C

compaired <- list(c(1,2),c(2,3),c(1,3))

data <- FetchData(scRNAsub2,vars = c("State","Exhausted_score1","CellType"))

data <- subset(data,CellType=="CD4 Tex")

p7.3.2 <- ggplot(data,aes(x=State,y=Exhausted_score1,fill=State))+geom_violin()+theme_classic()+ggtitle("Exhaustion score of CD4 Tex")+scale_x_discrete("")+

theme(title = element_text(face = "plain",size = 12),

#legend.title = element_blank(),

legend.text = element_text(size = 12),

legend.key.size = unit(0.5, 'cm'), #change legend key size

aspect.ratio = 1.2,

axis.text.x = element_blank(),

axis.text.y = element_text(size = 10),

axis.title.x = element_blank(),

axis.title.y = element_blank(),

panel.border = element_blank(),

axis.ticks.x = element_blank(),

plot.title=element_text(hjust = 0.5,vjust = 5))+

geom_boxplot(width=0.2,position=position_dodge(0.9),outlier.colour = NA,fill="white")+

geom_signif(comparisons = compaired,

step_increase = 0.2,

map_signif_level = T,

test = wilcox.test,textsize = 3.5)

p7.3.2

ggsave(p7.3.2,filename = 'FigureS4_C.png',width = 4,height = 5,dpi = 1000)

###########D

data <- FetchData(scRNAsub,vars = c("State","Exhausted_score1","CellType"))

data <- subset(data,CellType=="CD8 Tex")

p7.3.1 <- ggplot(data,aes(x=State,y=Exhausted_score1,fill=State))+geom_violin()+theme_classic()+ggtitle("Exhaustion score of CD8 Tex")+scale_x_discrete("")+

theme(title = element_text(face = "plain",size = 12),

#legend.title = element_blank(),

legend.text = element_text(size = 12),

legend.key.size = unit(0.5, 'cm'), #change legend key size

aspect.ratio = 1.2,

axis.text.x = element_blank(),

axis.text.y = element_text(size = 10),

axis.title.x = element_blank(),

axis.title.y = element_blank(),

panel.border = element_blank(),

axis.ticks.x = element_blank(),

plot.title=element_text(hjust = 0.5,vjust = 5))+

geom_boxplot(width=0.2,position=position_dodge(0.9),outlier.colour = NA,fill="white")+

geom_signif(comparisons = compaired,

step_increase = 0.2,

map_signif_level = T,

test = wilcox.test,textsize = 3.5)

p7.3.1

ggsave(p7.3.1,filename = 'FigureS4_D.png',width = 4,height = 5,dpi = 1000)

############FigureS5#################

###########A

BEAM_res2=BEAM_res2[,c("gene_short_name","pval","qval")]

BEAM_genes2 <- top_n(BEAM_res2, n = 100,dplyr::desc(qval)) %>% pull(gene_short_name) %>% as.character()

tmp2=plot_genes_branched_heatmap(mycds2[BEAM_genes2,],

branch_point = 1,

num_clusters = 3,

cores = 8,

branch_labels = c("State 3", "State 2"),

#hmcols = NULL,

#hmcols = colorRampPalette(rev(brewer.pal(9, "PRGn")))(62),

#hmcols = colorRampPalette(c("navy","white", "firebrick3"))(62),

branch_colors = c("#F8766D","#619CFF","#00BA38"),

use_gene_short_name = T,

show_rownames = T,

scale_max = 3,

scale_min = -3,

cluster_rows = T,

return_heatmap = T

)

png("FigureS5_A.png",width = 30*100,height = 35*100,res = 6*100)

tmp2$ph_res

dev.off()

gene_State2=tmp2$annotation_row

gene_State2$gene=rownames(gene_State2)

allcluster_go2=data.frame()

for (i in unique(gene_State2$Cluster)) {

small_gene_State=filter(gene_State2,gene_State2$Cluster==i)

df_name=bitr(small_gene_State$gene, fromType="SYMBOL", toType=c("ENTREZID"), OrgDb="org.Hs.eg.db")

go <- enrichGO(gene = unique(df_name$ENTREZID),

OrgDb = org.Hs.eg.db,

keyType = 'ENTREZID',

ont = "BP",

pAdjustMethod = "BH",

pvalueCutoff = 0.05,

qvalueCutoff = 0.2,

readable = TRUE)

go_res=go@result

if (dim(go_res)[1] != 0) {

go_res$cluster=i

allcluster_go2=rbind(allcluster_go2,go_res)

}

}

#allcluster_go$p.adjust <- round(allcluster_go$p.adjust,2)

head(allcluster_go2[,c("ID","Description","qvalue","cluster")])

###########B

#####CD4_EFS

fit1 <- survfit(Surv(EFS,EFS_event) ~State1, data = CD4_survival)

survival1<- ggsurvplot(fit1,

data = CD4_survival,

risk.table =F,

pval = F,

conf.int= F,

risk.table.height = 0.5,

legend.title = "",

legend.lab=c("high","low"),

legend = c(0.9,0.75),

#legend.labs = levels(lung[[i]]),##

surv.median.line = "hv",

palette="lancet",

pval.coord = c(2500,0.9))

plot1 <- survival1$plot+ylab("EFS")+xlab("Days")+ggtitle("State1")+theme_kcl+theme(axis.title.x = element_blank(),

plot.title = element_text(size = 14,hjust = 0.5,vjust = 0))+NoLegend()+annotate("text", x = 3000, y = 0.9, label = "p = 0.17",fontface=3,size=5)

fit2 <- survfit(Surv(EFS,EFS_event) ~State2, data = CD4_survival)

survival2<- ggsurvplot(fit2,

data = CD4_survival,

risk.table =F,

pval = F,

conf.int= F,

risk.table.height = 0.5,

legend.title = "",

legend.lab=c("high","low"),

legend = c(0.9,0.75),

#legend.labs = levels(lung[[i]]),##

surv.median.line = "hv",

palette="lancet",

pval.coord = c(2500,0.9))

plot2 <- survival2$plot+ylab("EFS")+xlab("Days")+theme_kcl+ggtitle("State2")+

theme(axis.text.y = element_blank(),axis.title.y = element_blank(),axis.ticks.y = element_blank(),

plot.title = element_text(size = 14,hjust = 0.5,vjust = 0))+NoLegend()+annotate("text", x = 3000, y = 0.9, label = "p = 0.067",fontface=3,size=5)

fit3 <- survfit(Surv(EFS,EFS_event) ~State3, data = CD4_survival)

survival3<- ggsurvplot(fit3,

data = CD4_survival,

risk.table =F,

pval = F,

conf.int= F,

risk.table.height = 0.5,

legend.title = "",

legend.lab=c("high","low"),

legend = c(0.9,0.75),

#legend.labs = levels(lung[[i]]),##

surv.median.line = "hv",

palette="lancet",

pval.coord = c(2500,0.9))

plot3 <- survival3$plot+ylab("EFS")+xlab("Days")+theme_kcl+ggtitle("State3")+

theme(axis.text.y = element_blank(),axis.title.y = element_blank(),axis.ticks.y = element_blank(),axis.title.x = element_blank(),

plot.title = element_text(size = 14,hjust = 0.5,vjust = 0))+NoLegend()+annotate("text", x = 3000, y = 0.9, label = "p = 0.005",fontface=3,size=5)

###CD4_OS

fit4 <- survfit(Surv(OS,OS_event) ~State1, data = CD4_survival)

survival4<- ggsurvplot(fit4,

data = CD4_survival,

risk.table =F,

pval = F,

conf.int= F,

risk.table.height = 0.5,

legend.title = "",

legend.lab=c("high","low"),

legend = c(0.9,0.75),

#legend.labs = levels(lung[[i]]),##

surv.median.line = "hv",

palette="lancet",

pval.coord = c(2500,0.9))

plot4 <- survival4$plot+ylab("OS")+xlab("Days")+theme_kcl+theme(axis.title.x = element_blank())+NoLegend()+annotate("text", x = 3000, y = 0.9, label = "p = 0.1",fontface=3,size=5)

fit5 <- survfit(Surv(OS,OS_event) ~State2, data = CD4_survival)

survival5<- ggsurvplot(fit5,

data = CD4_survival,

risk.table =F,

pval = F,

conf.int= F,

risk.table.height = 0.5,

legend.title = "",

legend.lab=c("high","low"),

legend = c(0.9,0.75),

#legend.labs = levels(lung[[i]]),##

surv.median.line = "hv",

palette="lancet",

pval.coord = c(2500,0.9))

plot5 <- survival5$plot+ylab("OS")+xlab("Days")+theme_kcl+

theme(axis.text.y = element_blank(),axis.title.y = element_blank(),axis.ticks.y = element_blank())+NoLegend()+annotate("text", x = 3000, y = 0.9, label = "p = 0.12",fontface=3,size=5)

fit6 <- survfit(Surv(OS,OS_event) ~State3, data = CD4_survival)

survival6<- ggsurvplot(fit6,

data = CD4_survival,

risk.table =F,

pval = F,

conf.int= F,

risk.table.height = 0.5,

legend.title = "",

legend.lab=c("high","low"),

legend = c(0.9,0.75),

#legend.labs = levels(lung[[i]]),##

surv.median.line = "hv",

palette="lancet",

pval.coord = c(2500,0.9))

plot6 <- survival6$plot+ylab("OS")+xlab("Days")+theme_kcl+

theme(axis.text.y = element_blank(),axis.title.y = element_blank(),axis.ticks.y = element_blank(),axis.title.x = element_blank())+

NoLegend()+annotate("text", x = 3000, y = 0.9, label = "p = 0.17",fontface=3,size=5)

(plot1+plot2+plot3)/(plot4+plot5+plot6)

ggsave((plot1+plot2+plot3)/(plot4+plot5+plot6),filename = "FigureS5_B.png",width = 9,height = 6.5,dpi = 1000)
